# Prediction and Inferred Evolution of Acid Tolerance Genes in the Biotechnologically Important *Acidihalobacter* Genus

**DOI:** 10.3389/fmicb.2022.848410

**Published:** 2022-04-18

**Authors:** Katelyn Boase, Carolina González, Eva Vergara, Gonzalo Neira, David Holmes, Elizabeth Watkin

**Affiliations:** ^1^Curtin Medical School, Curtin University, Perth, WA, Australia; ^2^Center for Bioinformatics and Genome Biology, Centro Ciencia & Vida, Santiago, Chile; ^3^Facultad de Medicina y Ciencias, Universidad San Sebastián, Santiago, Chile

**Keywords:** polyextremophile, extreme acidophile, acid resistance, genome evolution, phylogenomics, potassium transporters, urease, chloride/proton antiporters

## Abstract

*Acidihalobacter* is a genus of acidophilic, gram-negative bacteria known for its ability to oxidize pyrite minerals in the presence of elevated chloride ions, a capability rare in other iron-sulfur oxidizing acidophiles. Previous research involving *Acidihalobacter* spp. has focused on their applicability in saline biomining operations and their genetic arsenal that allows them to cope with chloride, metal and oxidative stress. However, an understanding of the molecular adaptations that enable *Acidihalobacter* spp. to thrive under both acid and chloride stress is needed to provide a more comprehensive understanding of how this genus can thrive in such extreme biomining conditions. Currently, four genomes of the *Acidihalobacter* genus have been sequenced: *Acidihalobacter prosperus* DSM 5130^T^, *Acidihalobacter yilgarnensis* DSM 105917^T^, *Acidihalobacter aeolianus* DSM 14174^T^, and *Acidihalobacter ferrooxydans* DSM 14175^T^. Phylogenetic analysis shows that the *Acidihalobacter* genus roots to the Chromatiales class consisting of mostly halophilic microorganisms. In this study, we aim to advance our knowledge of the genetic repertoire of the *Acidihalobacter* genus that has enabled it to cope with acidic stress. We provide evidence of gene gain events that are hypothesized to help the *Acidihalobacter* genus cope with acid stress. Potential acid tolerance mechanisms that were found in the *Acidihalobacter* genomes include multiple potassium transporters, chloride/proton antiporters, glutamate decarboxylase system, arginine decarboxylase system, urease system, *slp* genes, squalene synthesis, and hopanoid synthesis. Some of these genes are hypothesized to have entered the *Acidihalobacter* via vertical decent from an inferred non-acidophilic ancestor, however, horizontal gene transfer (HGT) from other acidophilic lineages is probably responsible for the introduction of many acid resistance genes.

## Introduction

Environments that exhibit both acidic and saline conditions are relatively rare. There are few studies aimed at understanding acid stress responses in halophiles and these typically show that halophiles are unable to grow in low pH environments (Bowers and Wiegel, 2011; Moran-Reyna and Coker, 2014; He et al., 2016; Zammit and Watkin, 2016). For example, it has been hypothesized that chloride ions, present in saline conditions, can disrupt the reversed membrane potential of acidophiles making them potentially sensitive to acidification of their cytoplasm followed by subsequent cellular death (Suzuki et al., 1999).

*Acidihalobacter* is one of a limited number of genera of bacteria that has been shown to be an extreme acidophile (pH ≤ 3.0) and halophile (Minegishi, 2013; Zammit and Watkin, 2016) and is thus a polyextremophile. Currently four organisms belonging to the *Acidihalobacter* genus have been isolated and cultivated in laboratory conditions: *Acidihalobacter prosperus* DSM 5130^T^ from the geothermally heated seafloor at Porto di Levante, Vulcano, Italy; *Acidihalobacter aeolianus* DSM 14174^T^, and *Acidihalobacter ferrooxydans* DSM 14175^T^ from the hydrothermal pools at the Aeolian Islands, Vulcano Italy and *Acidihalobacter yilgarnensis* DSM 105917^T^ from an acidic saline lake drain in Western Australia (Huber and Stetter, 1989; Simmons and Norris, 2002; Zammit et al., 2009). Several studies have been conducted on the *Acidihalobacter* genus, understanding its response to chloride ions, osmotic stress, metal stress and oxidative stress (Dopson et al., 2016; Khaleque et al., 2018, 2019, 2020) but there has been limited focus on their mechanisms of survival at extremely low pH. The *Acidihalobacter* are interesting not only because they are deeply intriguing polyextremophiles but also because they are among the few organisms that can be used for copper recovery (bioleaching) from chalcopyrite (Khaleque et al., 2017a). Extreme acidophiles capable of oxidizing iron and sulfur have been of interest to the scientific and industrial communities regarding their biotechnological and biomining applications (Johnson and Schippers, 2017; Gumulya et al., 2018) and their potential in protein engineering (Parashar and Satyanarayana, 2018).

Acidophilic microorganisms are found in all three domains of life. When describing acid stress responses in microorganisms, it is useful to make a distinction between “extreme” acidophiles (pH opt < 3.0) and “moderate” acidophiles (pH opt between 3 and 5). Both share multiple acid tolerance mechanisms, however, extreme acidophiles have additional adaptations absent in moderate acidophiles (reviewed in Baker-Austin and Dopson, 2007). Both categories of acidophiles thrive in acidic conditions by maintaining a circumneutral cytoplasmic pH despite a large proton gradient compared to their environment. In contrast, another class of acidophiles, the so-called “amateur” acidophiles, e.g., *Helicobacter pylori*, survive by neutralizing their acidic environment (Foster, 2004; Baker-Austin and Dopson, 2007; Slonczewski et al., 2009; Quatrini and Barrie Johnson, 2016). The maintenance of a circumneutral cytoplasmic pH is achieved through multiple mechanisms including the generation of a reversed membrane potential, creating a positive charge, referred to as the Donnan potential. This chemi-osmotic barrier effectively repels positive ions from entering the cell, preventing the acidification of their cytoplasm and has been defined as the first line of defense (Vergara et al., 2020). The use of potassium transporters, importing K^+^ ions, are thought to be the most likely mechanism acidophiles use to generate this membrane potential (Cholo et al., 2015; Buetti-Dinh et al., 2016; Christel et al., 2018). The potassium channel transporters Kdp, Trk, and Kch have been identified in extreme acidophiles (Cholo et al., 2015; Buetti-Dinh et al., 2016; Christel et al., 2018; Vergara et al., 2020). Extreme acidophiles are also hypothesized to have membranes that are less permeable to protons through membrane adaptations (Yamauchi et al., 1993; Macalady et al., 2004; Chao et al., 2008; Mykytczuk et al., 2010). Hopanoids, spermidine, and starvation-inducible membrane-altering lipoproteins are all associated with conferring a higher resistance to proton permeability (Alexander and St John, 1994; Hommais et al., 2004; Jones et al., 2012). Second line of defense mechanisms (Vergara et al., 2020) describe processes that consume or expel protons that have entered the cell including buffering reactions, for example the glutamate decarboxylase system, the arginine decarboxylase system, spermidine synthesis, the urease system and Na^+^/H^+^ antiporters (Hommais et al., 2004; Richard and Foster, 2004; Zhao and Houry, 2010; Feehily and Karatzas, 2013; Mangold et al., 2013; Marcus and Scott, 2016). These mechanisms are commonly found in both extreme and moderate acidophiles. To deepen our understanding of acid stress response in the *Acidihalobacter* genus, a genome-wide comparison study was undertaken to generate an inventory of predicted acid resistance genes. Using phylogenomic approaches, we suggest possible events, including gene gain/loss and gene duplication, that led to the evolution of the acidophilic *Acidihalobacter* from an inferred non-acidophilic ancestor.

## Materials and Methods

### Genomes and Phylogenetic Analysis

The four currently published *Acidihalobacter* assembly sequences (GCF_000754095.2, GCF_001753245.1, GCF_001753165.1 and GCF_001975725.1) as well as the outgroup *Halothiobacillus neapolitanus* c2 (GCF_000024765.1) were downloaded from the National Center for Biotechnology Information (NCBI) GenBank genomic database in October 2019 (Pruitt et al., 2012). Phylogenetic analysis of four *Acidihalobacter* genomes in the context of the Chromatiales order was conducted using 52 NCBI reference proteomes of the Chromatiales order available from the GenBank database (Supplementary Table 1) in October 2019. PhyloPhlAn3 was used to construct a phylogenetic tree of the Chromatiales proteomes, using the Phylophlan database and diversity set to low (set of 400 conserved proteins) (Asnicar et al., 2020). Diamond (Buchfink et al., 2015) was used for mapping the database to the proteomes in study, MAFFT (Katoh and Standley, 2013) for the multiple sequence alignment and the maximum likelihood tree was constructed with IQTREE (Nguyen et al., 2015), using 1000 replicates, with the best suited evolutionary model proposed by IQTREE. The final tree was visualized using iTOL.^1^

### Prediction of Mobile Genetic Elements and Genome Islands

Mobile genetic elements (MGEs) were predicted and classified using TnpPred and ISsaga (Siguier et al., 2006; Varani et al., 2011; Riadi et al., 2012). Horizontal gene transfer events were predicted by HGTector (Waack et al., 2006; Zhu et al., 2014). Genome context of mobile elements and genome islands were analyzed using STRING, MAUVE, and ARTEMIS (Darling et al., 2010; Carver et al., 2012; Szklarczyk et al., 2019). These bioinformatic tools were used to predict genes involved in mechanisms of HGT (see Supplementary Information 1).

### Identification of Genes Related to Low pH Resistance

Genes and mechanisms involved in acidic resistance were identified through previous research (Vergara et al., 2020; González-Rosales et al., 2022). Identification of these genes in the *Acidihalobacter* genus genomes and the outgroup was done using BlastP comparison with a minimum E-value cutoff of 1e^–5^. Synteny blocks were predicted and visualized using MAUVE Progressive alignment (Darling et al., 2010). Genome contexts were visualized using Artemis (Carver et al., 2012). Acid tolerance proteins found in the *Acidihalobacter* genomes and *H. neapolitanus* were back-blast to assess orthologous proteins in other organisms. To assess the relatedness of these proteins, phylogenetic trees were made as previously described in section 2.1 using MAFFT, IQTREE, and iTOL.

### Mapping Evolutionary Events

To infer branch-site-specific evolutionary events across genomes of the *Acidihalobacter* genus, a conserved markers tree of 400 proteins was constructed between the *Acidihalobacter* genomes and using *H. neapolitanus* c2 as outgroup using Phylophlan3 (Asnicar et al., 2020). The presence of genes predicted to be involved in acid resistance were mapped onto each branch of the phylogenomic tree to model gene gain and loss events. Inference of evolutionary events were based on parsimony criteria.

## Results and Discussion

### Genomic Features of the Acidihalobacter Genus

*Acidihalobacter* spp. are aerobic, chemolithotrophic, mesophilic, halotolerant acidophiles with pH optimums between 1.9 and 2.75. The four publicly available *Acidihalobacter* genomes were analyzed together with the *H. neapolitanus* outgroup genome (Table 1). *A. yilgarnensis* is the only complete genome of the *Acidihalobacter*, with the other three being high quality permanent draft genomes. GC% content between the *Acidihalobacter* genomes varies between 59.9 and 64.4%. *Acidihalobacter* genomes are larger than the *H. neapolitanus* c2 outgroup genome with a range of 0.78–0.99 Mb.

**TABLE 1:** Genomic information of the *Acidihalobacter* spp. genomes and *Halothiobacillus neapolitanus* genome used in the study.

Genome	Size (Mb)	No. predicted genes	G+C (%)	Stat-us	RefSeq Assembly Accession	Origin	pH optima [range]	NaCl (M) optima [range]	Ref
*A. prosperus^T^* DSM 5130	3.36	3264	64.4	D	GCF_000754095.2	Italy	2 [1–4.5]	0.34 [0.07–1]	Nicolle et al., 2009; Ossandon et al., 2014
*A. yilgarnensis^T^* DSM 105917	3.57	3459	59.9	C	GCF_001753245.1	WA	2.5 [2–4]	0.4 [0.005–1.28]	Khaleque et al., 2017a
*A. aeolianus^T^* DSM 14174	3.36 (0.16)	3234 (165)	62.1 (57.3)	D (C)	GCF_001753165.1	Italy	1.8 [1.5–3]	0.4 [0.06–1.28]	Khaleque et al., 2017c
*A. ferrooxydans^T^* DSM 14175	3.45	3220	61.6	D	GCF_001975725.1	Italy	1.8 [1–3]	0.4 [0.06–0.85]	Khaleque et al., 2017b
*H. neapolitanus* c2 DSM 15147	2.58	2407	54.7	C	GCF_000024765.1	VA	6.5–6.9 [3-8.5]	0-0.86 [ND]	Boden, 2017; Kelly and Wood, 2000

*A. aeolianus is the only species to have a plasmid (termed pABPV6, displayed in parentheses). D, draft genome; C, closed (finished) genome. Square brackets show the range of pH and NaCl for growth. WA, Western Australia; VA, Victoria Australia.*

### Phylogeny of *Acidihalobacter* Within the Chromatiales Order

**FIGURE 1:**
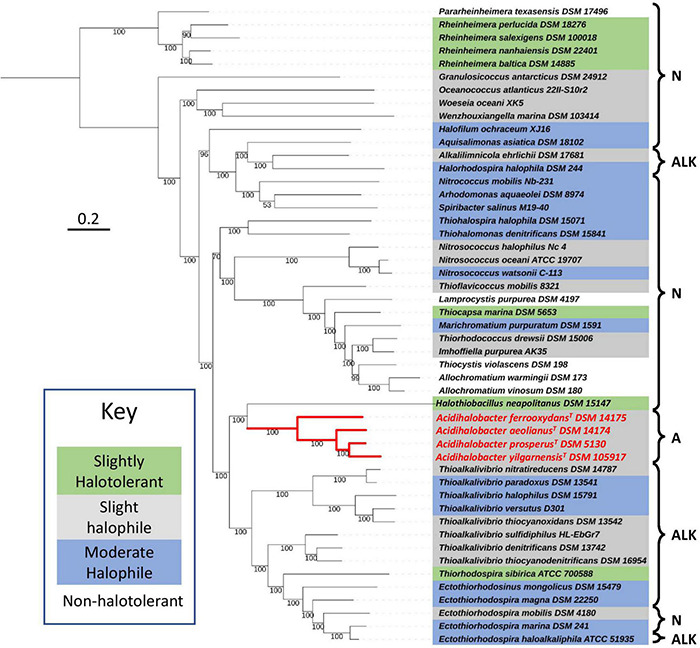
Phylogenomic tree constructed, using 400 protein markers of 52 representative complete genomes of the Chromatiales order, using the Phylophlan3 program. Gray highlights slightly halophilic genomes (optimum growth 2–5% w/v NaCl), blue highlights moderately halophilic genomes (optimum growth 5–20% w/v NaCl), green highlights slightly halotolerant genomes (no NaCl required to grow however optimum growth in 2–8% w/v NaCl) and no highlight represents non-halophiles. A, acidophiles (optimum growth < pH 5); N, neutrophiles (optimum growth between pH 5 and 9); and ALK, alkaliphiles (optimum growth > pH 9). Red lines indicate the phylogenetic relationships between the acidophiles. The sequences were aligned with MAFFT and the concatenated alignment was used to construct the phylogenetic tree with IQTREE and LG+R3 as the best-suited evolutionary model. Scale bar indicates 0.2 substitutions per amino acid. See Supplementary Table 1 for the full information on the microorganisms used in the tree.

To understand the recent evolutionary history of the *Acidihalobacter* genus, we compared the relative chloride and acid tolerance of microbes in the Chromatiales order to that of the *Acidihalobacter* spp. Using the complete reference genomes in the Chromatiales order, a phylogenetic tree was constructed, and literature was reviewed to identify the chloride and acid tolerances of the genomes used (Figure 1). Most microbes in the Chromatiales order were halotolerant and either neutrophilic or alkaliphilic. The *Acidihalobacter* microbes were the only microbes in the Chromatiales order that tolerate pH environments below four. This is an indication that *Acidihalobacter* has likely evolved from a halophilic past and has gained the ability to cope with acidic stress.

### Acid Resistance: First Line of Defense

“First line of defense” (Vergara et al., 2020) mechanisms are involved in reducing the influx of protons into the cytoplasm of acidophiles. Potassium pumps are primarily responsible for this by creating the reversed membrane potential, limiting proton influx (reviewed in Quatrini and Barrie Johnson, 2016). Membrane alterations that limit proton permeability across the cellular membrane also fall under the “first line of defense” mechanisms and include hopanoid synthesis, Slp proteins and porin alterations, as described in the following sections.

#### Membrane Potential and Potassium Transporters

Potassium transport systems are found in many bacteria as potassium is the major intracellular cation responsible for osmotic regulation, activating intracellular enzymes, regulating internal pH and as a secondary messenger (Epstein, 2003; Korolev, 2021; Raven, 2021). It is widely accepted that potassium transporters are regulated by ion concentrations and turgor pressure (Epstein, 2003). In terms of acid tolerance in extreme acidophiles, potassium transporters are hypothesized to be responsible for the reversed membrane potential, which is the primary mechanism reducing proton influx (Baker-Austin and Dopson, 2007). In halophiles, it has been shown that potassium transport systems aid in osmotic stress and typically halophiles have multiple copies of potassium transport genes (Epstein, 2003; Kraegeloh et al., 2005; Mongodin et al., 2005).

The Trk potassium system symports K^+^ and H^+^ into the cell and has been identified in plants, Bacteria and Archaea (reviewed in Epstein, 2003; Pandey and Mahiwal, 2020). In prokaryotes the most studied Trk system is in *Escherichia coli* and consists of TrkH and TrkG, the multimeric functional transporters; TrkA, the cytoplasmic regulating subunit; and TrkE (also known as *sapD*), the ATP-binding component of the Trk system (Epstein, 2003; reviewed in Stautz et al., 2021). In *E. coli*, the genes involved in the Trk system are distributed throughout the genome, however in halophilic organisms *trk*A and *trk*H have been found clustered together (Nakamura et al., 1998; Kraegeloh et al., 2005). Many acidophile genomes lack *trk*HG, and only contain the *trk*A homolog (exception *Alicyclobacillus ferrooxidans*) (Rivera-Araya et al., 2020). In *Acidihalobacter* spp. and *H. neapolitanus c2* the TrkAH potassium transporter system was present. The system is in a highly conserved syntenic region in all genomes and the genes are arranged contiguously (Supplementary Figure 1). A second copy of *trk*H was also found in the *Acidihalobacter* genomes directly downstream of the first *trk*H copy. The genomic arrangement of the Trk system in *Acidihalobacter* and *H. neapolitanus* c2 is very similar to that found in *Vibrio alinolyticus* (a halophile) with *fmt* (methionyl-tRNA formyltransferase) and *rsmB* (a ribosomal RNA small subunit methyltransferase B, also identified as *fmu* in other bacteria) being present upstream of *trk*A (Supplementary Figure 1) (Nakamura et al., 1998). Three extra genes were found between *rsm*B and *trk*A in *Acidihalobacter* and *H. neapolitanus*, a predicted gene that produces a protein with an unknown function, a histidine kinase, and a sigma-54 RNA polymerase holoenzyme. Both the histidine kinase and σ-54 proteins are predicted to be nitrogen regulated and involved in nitrogen assimilation (*ntr*Y and *ato*C also known as *ntr*X). Trk proteins have been identified in the genomes of other acidophiles such as *Leptospirillum* (Vergara et al., 2020), however, information about how the Trk system is arranged in their genome is lacking. No other case of a Trk system with two adjacent TrkH proteins has been published. Multiple copies of *trk*A and *trk*H have been found in the same region in *Salinibacter ruber*, however, they are not juxtaposed (López-Pérez et al., 2013). Whether this alteration results in a more efficient adaptation to acidity or salinity remains to be explored. Evidence of HGT in the genome surrounding the Trk system in *Acidihalobacter* was not found, and phylogenetic analysis of the protein sequences and their best BlastP hits show the *Acidihalobacter* TrkH proteins clustering with other TrkH proteins found in other organisms in the same Chromatiales order (Supplementary Figure 2). In light of the Trk system being in a highly conserved region of the genome in both *Acidihalobacter* and *H. neapolitanus* c2 and the Trk proteins all clustering with Trk proteins in the Chromatiales order, vertical descent of the Trk system in *Acidihalobacter* is likely. Whether the alteration of the gene structure in *Acidihalobacter* and *H. neapolitanus* when compared to Trk systems in other halophiles results in differential control or efficiency of Trk in these bacteria remains to be investigated.

Kdp is a high-affinity potassium transport system known to be involved in potassium homeostasis and the osmotic stress response in many bacteria, encompassing halophiles and non-halophiles, and is responsible for the active influx of potassium ions in environments with low potassium concentrations (Epstein, 2003; Strahl and Greie, 2008; Kixmüller et al., 2011; Price-Whelan et al., 2013). The Kdp system is also present in many acidophiles and has been found to be actively transcribed in acidic environments (Bakker et al., 1987; Acuña et al., 2013; Cholo et al., 2015; Rivera-Araya et al., 2019, 2020; Vergara et al., 2020). In *E. coli* the Kdp system consists of two operons, one encompassing the structural genes *kdp*FABC, and the other encompassing the regulatory genes *kdp*DE (Walderhaug et al., 1992). These two operons overlap in *E. coli*, however, different genetic arrangements of the system are found in other bacteria (Walderhaug et al., 1992; Epstein, 2003). KdpF is involved in stabilizing the Kdp complex but is not essential. KdpA is responsible for binding and translocating the K^+^ and KdpB and KdpC are responsible for the ATP hydrolysis (Hesse et al., 1984; Gaßel et al., 1998, 1999; Dorus et al., 2001; Reviewed in Epstein, 2003). The *kdp*DE operon is a two-component response regulator responsible for regulating the Kdp complex as KdpD is a histidine kinase and KdpE is a response regulator (Walderhaug et al., 1992; Reviewed in Epstein, 2003). The Kdp system was only identified in the genome of *A. aeolianus.* The gene arrangement of the system was similar to that of *E. coli* (Supplementary Figure 3A) (Walderhaug et al., 1992). Both carbonic anhydrase (*can*) and the sigma-54 regulator (σ-54) have been identified in the same genomic context and previously have been indicated to be involved in acid tolerance of other microorganisms (Sachs et al., 2006; Bury-Moneì et al., 2008; Mitra et al., 2012, 2014). HGTector analysis predicts KdpA, KdpB, KdpC, KdpD, KdpE, and the sigma-54 transcription regulator to have been gained through HGT from a Bacteria, and the *can* from a Proteobacteria. The sigma-54 regulator, a hypothetical zinc-containing protein and the *can* upstream of the Kdp system all have significant similarity to their corresponding proteins in multiple *Acidithiobacillus* spp. (*Acidithiobacillus ferriphilus* and *Acidithiobacillus ferrivorans*) as well as the closely related *Ambacidithiobacillus sulfuriphilus* and *Fervidacidithiobacillus caldus* acidophiles. The lack of *kdp* genes in all other *Acidihalobacter* genomes and the outgroup suggests it has been acquired via HGT, as well as the genes and gene contexts having significant similarity to species in the Burkholderiales order. We propose that the Kdp system in *A. aeolianus* was a result of HGT and has the potential to aid acid tolerance in low potassium environments.

Kch is a voltage-gated membrane potassium transporter commonly found in plants but has also been identified in *E. coli*, although its biological role is currently not understood (Reviewed in Epstein, 2003; Lundbäck et al., 2009). In eukaryotes, it is recognized as a voltage-gated ion channel that is associated with membrane potential regulation (Capera et al., 2019). The Kch protein was identified in all *Acidihalobacter* genomes and was not identified in *H. neapolitanus*. Best-hit analysis of the Kch proteins to the NCBI BlastP non-redundant database revealed that the Kch proteins in *Acidihalobacter* have significant similarity (46–53%) to Kch proteins from the acidophiles, *F. caldus* and *Am. sulfuriphilus*. *put*A (a bifunctional proline dehydrogenase/L-glutamate gamma-semialdehyde dehydrogenase) was found closely associated with *kch* in *A. yilgarnensis* and *A. prosperus* and is known to be involved in osmotolerance. Phylogenetic analysis, however, shows the Kch proteins from *Acidihalobacter* clustering closely to Kch proteins from other Chromatiales, and not the acidophiles Kch proteins (see Supplementary Figure 4). As Kch proteins are present, and cluster closely to the Chromatiales proteins, it is likely the Kch protein is a result of vertical decent in the *Acidihalobacter* genomes. The proximity of the *kch* to other osmotic tolerance genes may point to this area of the genome being important to both acid and osmotolerance in *Acidihalobacter.*

#### Spermidine Biosynthesis and Associated Genes

**FIGURE 2:**
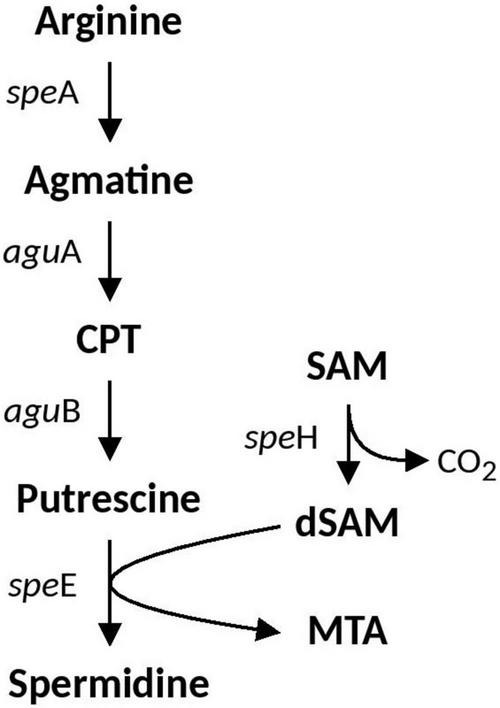
Spermidine biosynthesis pathway identified in *Acidihalobacter.* CPT, N-carbamoylputrescine; SAM, S-Adenosyl methionine; dSAM, decarboxylated-Adenosyl methionine; MTA, methylthioadenosine.

Spermidine is a positively charged membrane associated aliphatic, polycation polyamine that is possibly involved in acid and osmotic stress responses in *E. coli* and has been shown to reduce the outer membrane permeability caused by porins (Samartzidou et al., 2003). Spermidine biosynthesis pathways vary between bacterial species. Arginine is the precursor which can either be converted to citrulline or ornithine (via ArcB and RocF, respectively) which is then converted to putrescine (via SpeC) and subsequently converted to Spermidine via SpeE. An alternative pathway also includes arginine conversion to agmatine (AguA), which can be either converted directly to putrescine via SpeB, or to an intermediate CPT by AguA. The full spermidine biosynthesis pathway identified in *Acidihalobacter* is summarized in Figure 2. Both *spe*E and *spe*H (involved in the production of dSAM important in the conversion of putrescine to spermidine) were identified in all *Acidihalobacter* genomes and had significant similarity (66–68%) to SpeE and (73–79%) SpeH proteins in the closely related sister clade *Thioalkalivibrio* genus and other bacteria in the Chromatiales order. *spe*E is found in the same genomic context as the urease accessory genes. *spe*H is in a separate part of the genome that is also conserved between the *Acidihalobacter* genomes. Genes involved in osmotic tolerance are found upstream of *spe*H including *yhf*A (a protein belonging to the osmotically inducible OsmC superfamily). As spermidine has been shown to be involved in both acid and osmotic stress responses, it is likely that spermidine synthesis in *Acidihalobacter* has been acquired via vertical descent from a common ancestor considering the presence of spermidine genes in other organisms from Chromatiales order and has possibly been lost in the *H. neapolitanus* outgroup genome.

#### Hopanoid Biosynthesis

**FIGURE 3:**
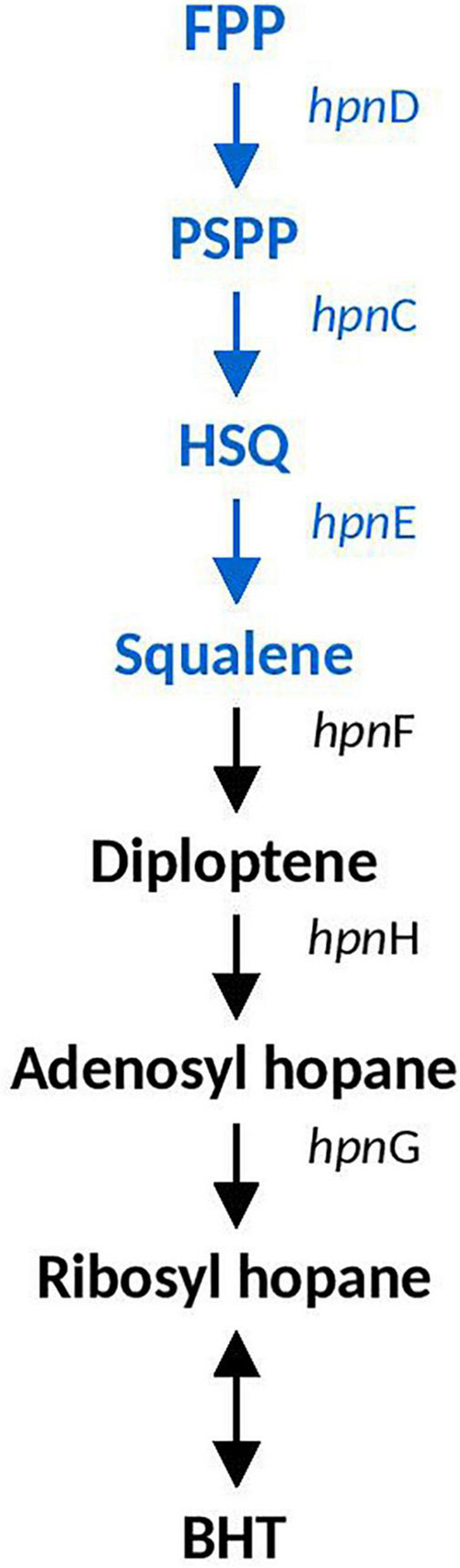
Hopanoid biosynthesis pathway identified in *Acidihalobacter.* Blue text indicates pathway identified in all *Acidihalobacter* genomes, black text indicates hopanoid biosynthesis pathway found in *A. ferrooxydans.* HSQ, hydroxysqualene; BHT, bacteriohopanetetrol.

Hopanoid biosynthesis is hypothesized to be involved in regulating the fluidity and permeability of bacterial membranes, aiding in stress responses involved in acidity, temperature and salinity (Belin et al., 2018). Squalene is the precursor to hopanoid biosynthesis and the genes involved in it (*hpn*DE and *hpn*C) were predicted in all *Acidihalobacter* genomes, and were not identified in the *H. neapolitanus* genome. *hpn*DEC all had high similarity to orthologous proteins in the *Thioalkalivibrio* genus (>90%). *A. ferrooxydans* was the only genome that had the complete set of genes required to produce bacteriohopanetetrol (BHT) (*hpn*FHG) (the full hopanoid biosynthesis pathway can be seen in Figure 3). HpnF and HpnH both had high similarity to proteins from the *Nitrosomonas* genus (61.5 and 74–68%, respectively) and HpnH also had high similarity to proteins from microbes in the Methylococcaceae family (70–67%). *hpn*H, *isp*H, *hpn*F, *hp*G, and *hpn*A are all in a region of the genome with no synteny with the other *Acidihalobacter* genomes. An assembly gap was detected in the genome adjacent to *hpn*A, thus the full genomic context and evidence of HGT could not be determined.

#### Starvation Lipoprotein Slp

**FIGURE 4:**
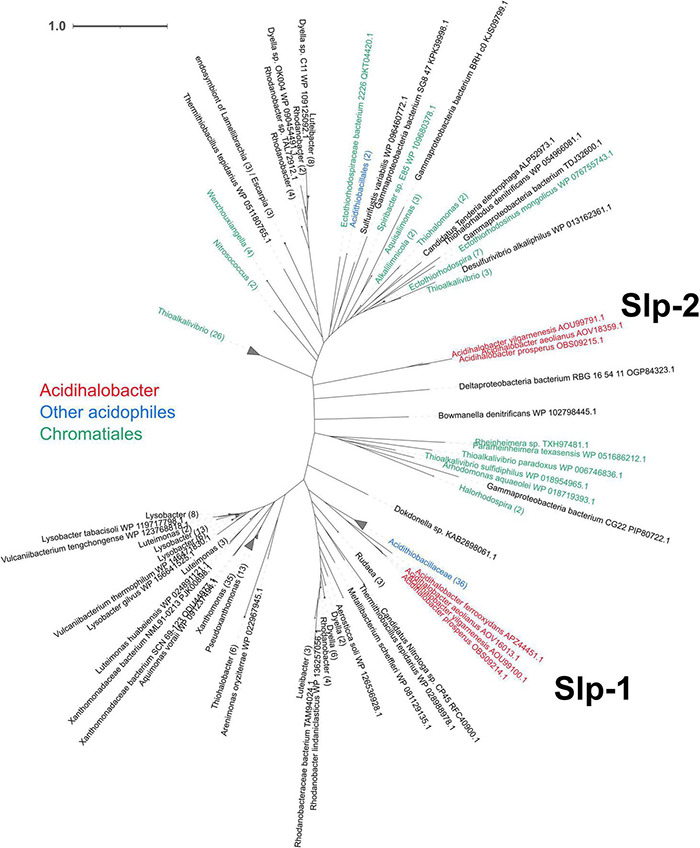
Unrooted phylogenetic tree of the predicted *Acidihalobacter* Slp amino acid sequences and their best hits from the NCBI non-redundant database, with collapsed branches by genera when possible (showing in parenthesis the number of leaves inside each). *Acidihalobacter* Slp proteins are colored red, Chromatiales proteins are colored green and other acidophiles proteins are colored blue. The time scale bar represents 1 amino acid substitution per site.

The outer membrane “starvation lipoprotein” gene *slp* is associated with the protection of *E. coli* during carbon starvation and metabolites that are toxic in low pH environments (namely organic acids) (Alexander and St John, 1994; Hommais et al., 2004; Mates et al., 2007). *slp* is located in the “acid fitness island” of *E. coli* and is co-expressed with other acid resistance genes. Two copies of *slp* were identified in all species of *Acidihalobacter*, excluding *A. ferrooxydans*, which only had one copy, and was absent in the outgroup. Slp copies contained a characteristic lipobox motif (Zückert, 2014; Vergara et al., 2020) with an Alanine amino acid residue in the position +2, suggesting an exportation of Slp (Supplementary Figure 5). Both copies, named *slp-1 and slp-2*, are adjacent to each other in *A. yilgarnensis, A. prosperus and A. aeolianus*, and the area of the genome is conserved between the three species (see Supplementary Figure 6). A cardiolipin synthase (*cls*) gene was found upstream of *slp* in all *Acidihalobacter* genomes and is involved in the structural organization of membranes and is associated with osmoregulation and acidic resistance (Romantsov et al., 2009; MacGilvray et al., 2012). The cardiolipin synthase presented higher similarity with Planctomycetes microorganisms according BlastP results. *engB*, a gene associated with the *slp* and glutamate decarboxylase genes in *E. coli*, was also identified in the same genomic context as *Acidihalobacters slp* (Mates et al., 2007). *NhaP*, a K^+^(Na^+^)/H^+^ antiporter, and *lysR*, a transcriptional regulator, were identified downstream of *slp* in *A. ferrooxydans* and both are associated with acid stress response of *Vibrio cholerae* (Kovacikova et al., 2010; Ante et al., 2015; Mourin et al., 2019). Phylogenetic analysis of the proteins shows the Slp-1 protein clustering with Slp proteins of *Acidithiobacillus*, and Slp-2 clustered with Chromatiales Slp proteins (see Figure 4). HGT events signals were identified for two genes upstream of slp-1 (AOU99095.1–AOU99096.1) by HGTector, suggesting Proteobacteria donor. This information, in addition to the genomic arrangement and phylogenetic analysis, allow us to hypothesize that genomic segment of *Acidihalobacter* including *slp-*1, cardiolipin synthase and genes upstream were gained by HGT events from a Proteobacteria donor, such as the extreme acidophile *Acidithiobacillaceae*; meanwhile *slp-*2 copies correspond to an ancestral gene from Chromatiales.

### Acid Resistance Mechanisms: Second Line of Defense

“Second line of defense” (Vergara et al., 2020) mechanisms are involved in removing excess protons that have passed the cellular membrane and have entered the cytoplasm. Such mechanisms consist of cytoplasmic buffering systems and proton antiporters, pumping protons out of the cell. The glutamate decarboxylase system, the arginine decarboxylase system, the urease system, carbonic anhydrase and the ClcA antiporters found in *Acidihalobacter* are described below.

#### Glutamate Decarboxylase System

The glutamate decarboxylase system has been identified as a major mechanism in the acid stress response of *E. coli* (reviewed in Foster, 2004). Glutamate is transported into the cell by GadC (an amino acid permease), and GadA or GadB (the glutamate decarboxylase isozymes) replaces the *a*-carboxyl group of glutamate with H^+^, creating *y*-amino butyric acid (GABA) and CO_2_ removing a proton from the cytoplasm in the process. GABA is subsequently removed from the cell by GadC. The Gad decarboxylase system has also been identified in extreme acidophiles such as *Leptospirillum* spp. and *F. caldus* (Mangold et al., 2013; Vergara et al., 2020). GadA and GadC were both identified in all *Acidihalobacter* genomes but was missing in the *H. neapolitanus* genome. *gad*A and *gad*C in *A. aeolianus* and *A. ferrooxydans* were separated by a protein with no predicted putative domains. This hypothetical protein was predicted to be 248 amino acids and 7 transmembrane domains by HMMER, and has matches to ArcD, an arginine/ornithine antiporter in the HMMER database. In all genomes, best-hit analysis of GadA and GadC proteins showed 100% coverage and high amino acid percentage identity (ranging from 75 to 85.12%) to orthologs in *Salinisphaera* sp. LB1 (a haloacidophile), *Mangrovitalea sediminis* (a halophile), and *Acidithiobacillus* spp. (including *At. thiooxidans, At. Ferridurans*, and *At. ferrivorans*). This is an indication of a possible gene gain event in *Acidihalobacter* (see Supplementary Figures 7A,B).

#### Arginine Decarboxylase System

The Arginine decarboxylase system is another acid stress tolerance system identified in *E. coli* that works in the same way as the glutamate decarboxylase system (reviewed in Richard and Foster, 2004). Arginine decarboxylase, encoded by the *adiA*, replaces the *a*-carboxyl group of arginine with a proton, producing CO_2_ and agmatine, which is removed from the cell via antiporter AdiC. All *Acidihalobacter* genomes had hits to *adiA*, however, no hit to *adiC* was identified (no arginine decarboxylase genes were found in the *H. neapolitanus* genome). The complete arginine synthesis pathway was identified (*arg*ABCDEFGHO), thus *Acidihalobacter* may be capable of producing its own arginine, to be used in the arginine decarboxylase system. Best hits of the *Acidihalobacter* AdiA proteins to the BlastP non-redundant database indicated significant similarity to AdiA proteins of other Chromatiales organisms, suggesting vertical descent.

#### Urease System

One of the key mechanisms used by the enteric pathogen *H. pylori* to combat acid stress is the production of a highly expressed urease enzyme (Marcus and Scott, 2016). Urease converts urea to ammonia which is protonated to NH_4_^+^, neutralizing its environment. Urea import in *H. pylori* is controlled by UreI, a proton gated inner membrane urea channel, found to be activated by low pH (Bury-Moné et al., 2001; McNulty et al., 2013). Further buffering capacity is achieved by carbonic anhydrase which converts the carbon dioxide produced as a by-product of the urease activity into carbonic acid, which has a higher buffering capacity compared to ammonium (Marcus et al., 2005). The urease enzyme is coded for by *ure*A, *ure*B, and *ure*C (urease subunit gamma, beta, and alpha, respectively) and *ure*D (an ortholog of *ure*H), *ure*E, *ure*F, and *ure*G all of which are accessory proteins necessary for the expression of a functional urease enzyme (Akada et al., 2000). In *H. pylori* the regulation in the expression of the urease complex is dependent on the cellular pH and environmental pH, conditions sensed by the ArsRS and FlgRS two-component system. Urease is also found widely in soil bacteria, cyanobacteria and the extreme acidophile *Ferrovum* (Ullrich et al., 2016a) and studied in halophilic alkaliphiles in the production of biocement (Burbank et al., 2012; Dhami et al., 2013; Stabnikov et al., 2013; Veaudor et al., 2019). In the previously mentioned cases the urease activity is largely related to nitrogen assimilation and microbially induced calcite precipitation (Burbank et al., 2012). In cyanobacteria urea transport is controlled by the urea transport genes *urt*ABCDE (Veaudor et al., 2019).

**FIGURE 5:**
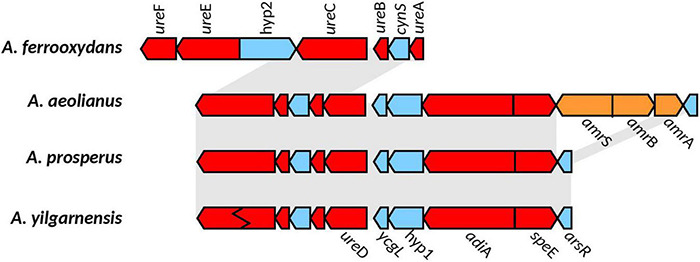
Genomic context of the urease gene cluster in the *Acidihalobacter* spp. genomes. Gray background shows synteny between genomes; blue genes are found in all genomes; red shows genes hypothesized to be involved in acid and osmotic tolerance; genes in orange correspond to unique genes (only found in their respective genomes). Ziz-zag pattern indicates a frameshift mutation in the gene. Hyp1, hypothetical 1; Hyp2, hypothetical 2; *cyn*S, cynanate hydratase; *ycg*L, protein YcgL; *ars*R, arsenic resistance transcriptional regulator; *amr*S, AmmeMemoRadiSam system radical SAM enzyme; *amr*B, AmmeMedoRadiSam system protein B; *amr*A, AmmeMedoRadiSam system protein A.

In all the *Acidihalobacter* genomes, *ure*A, *ure*B, *ure*C, *ure*D, *ure*E, *ure*F, and *ure*G were detected (*ure*C in *A. yilganensis* has multiple frameshift mutations resulting in a truncated version of UreC). These genes were not detected in the outgroup *H. neapolitanus* c2. The gene cluster structure of the urease system was unique in the *Acidihalobacter* genomes with a cyanase (*cyn*S) gene between *ure*A and *ure*B. Cyanase is an enzyme that converts cyanate to carbamate, which spontaneously converts to ammonia and carbon dioxide (OCN^–^ + HCO_3_^–^ + 2H^+^ ↔ NH_3_ + 2CO_2_), directly consuming two protons as well as more protons consumed via the spontaneous conversion of ammonia to ammonium (NH_3_ + H^+^ ↔ NH_4_^+^) (Anderson and Little, 1986). Reporting of *cyn*S incorporated into the urease operon in other genomes has not been found and could possibly be a novel alteration in *Acidihalobacter* allowing for further buffering capacity. As previously stated, *H. pylori* contains *ars*R, a pH activated transcriptional regulator, responsible for the transcription of the urease operon in low pH environments (Pflock et al., 2005). The *ars*R transcriptional regulator was found in *A. yilgarnensis, A. prosperus*, and *A. aeolianus.* This regulator was found upstream of the urease operon and also encompasses the genes *adi*A and *spe*E (Figure 5). SpeE is a polyamine aminopropyltransferase in *E. coli* that is involved in the production of spermidine and is found to be involved in the acid tolerance of *E. coli*, possibly through the regulation of a wide range of acid tolerance genes including the glutamate decarboxylase system and the arginine decarboxylase system (Rhee et al., 2007). Whether or not the urease operon, *adi*A and *spe*E are controlled by the *ars*R transcriptional regulator remains to be seen and must be tested further. The only urease transport system detected was *urt*ABCDE in *A. ferrooxydans* and two copies in *H. nepolitanus* c2. In the case of *H. pylori*, gastrointestinal urea concentrations are very low (∼3 mM), and thus requires the active transport of urea into its cell for the urease system to be effective at combating acid stress (Scott et al., 2000). Environmental urea typically ranges from 0 to 13uM in concentration, over 2 orders of magnitude lower than the case of *H. pylori*; thus, it is unclear if the urease system found in *A. yilgarnensis, A. prosperus* and *A. aeolianus* would be effective enough in combating acid stress if no active urea transport system is found. *A. ferrooxydans* may be able to use the urease system as an acid resistance mechanism more effectively than the other member of *Acidihalobacter* due to the presence of the active urease transporter system in its genome. Further proteomic studies would be needed to validate this hypothesis. Due to the lack of the urease gene cluster in the outgroup and the similarity of the *Acidihalobacter* and Betaproteobacteria urease gene clusters, we speculate that the urease system was acquired via HGT in an ancestral *Acidihalobacter*.

#### Carbonic Anhydrase

Carbonic anhydrase (CA) is a proton-consuming enzyme that has been found to be upregulated in response to acid stress in *H. pylori* and the lack of the enzyme delays growth of *H. pylori* in acidic conditions (Sachs et al., 2006; Bury-Moneì et al., 2008). CAs are found in the cytoplasm and thus would directly contribute to minimizing the effect of protons leaking into the cytoplasm. CA is also known to catalyze the dissolution of carbonate minerals and in *Aspergillus fumigatus* is upregulated in potassium limited environments to increase the dissolution of potassium minerals to obtain potassium (Xiao et al., 2012; Sun et al., 2013). CA have also been found in acidophiles, but conclusions about their involvement in acid tolerance is not clear (Levicán et al., 2008; Li et al., 2012; Acuña et al., 2013). HGTector predicted the CA gene to have been acquired in the *A. aeolianus* and the *A. yilgarnensis* genomes from a Proteobacteria. The proximity of the CA to the Kdp system in *A. aeolianus*, as well as its clustering with CA proteins from the extreme acidophile class *Acidithiobacillia* may indicate an involvement in acid tolerance. Although, best BlastP hit analysis had many top hits to CAs of the extreme acidophile class *Acidithiobacillia*, phylogenetic analysis reveals clustering of the *Acidihalobacter* CAs to both *Acidithiobacillia and Chromatiales* CAs (see Supplementary Figure 8). This makes it unclear as to whether the horizontal gene transfer occurred from the acidophiles to the *Acidihalobacter* genomes, and subsequent loss in *A. prosperus* and *A. ferrooxydans*, or if the HGT occurred from an early *Acidihalobacter* common ancestor to the acidophiles.

#### ClcA Antiporters

**FIGURE 6:**
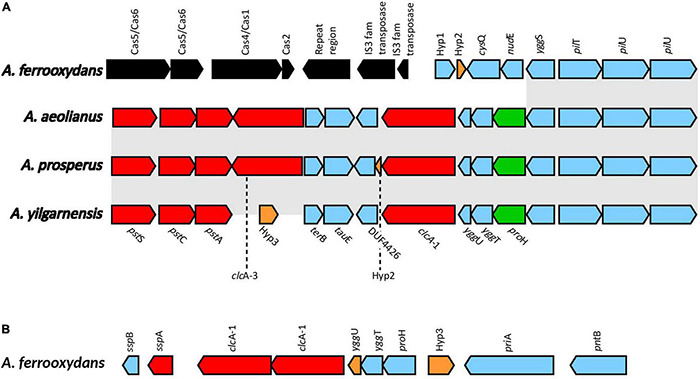
**(A)** The genomic context of *clc*A-1 in *A. yilgarnensis, A. prosperus* and *A. aeolianus*. **(B)** The genomic context of the *clcA-1* genes in *A. ferrooxydans.* Genes in red; involved in acid tolerance, in green; involved in osmotolerance, in blue; others, in black; genes associated with HGT. Hyp, hypothetical proteins with no predicted putative domains; *cys*Q, 3′(2’),5′-bisphosphate nucleotidase; *nud*E, ADP compounds hydrolase; *ygg*S, Pyridoxal phosphate homeostasis protein; *pil*T, type IV pilus twitching motility protein; *pil*U, type IV pilus ATPase; *ter*B, tellurite resistance protein; *tau*E, sulfite exporter; DUF4426, domain of unknown function 4426; *ygg*U, UPF0235 protein YggU; *ygg*T, uncharacterized protein; *pro*H, pyrroline-5-carboxylate reductase 1; *ssp*B, stringent starvation protein B; *ssp*A, stringent starvation protein A; *pri*A, primosomal protein N′; *pnt*B, NAD(P) transhydrogenase subunit beta.

Voltage gated ClC-type Cl^–^/H^+^ transporters found in *E. coli* are thought to be important in acid resistance systems. These proteins are hypothesized to function as a “electrical shunt for an outwardly directed virtual proton pump, linked to amino acid decarboxylation” (Iyer et al., 2002). Although chloride regulation is important in halophiles, no studies have indicated ClcA proteins to be involved in osmotic tolerance. Multiple predicted ClcA proteins were identified in the *Acidihalobacter* genomes, two in *A. yilgarnensis*, three in *A. prosperus* and *A. aeolianus*, four in *A. ferrooxydans* and one in the outgroup. Phylogenetic analysis of the ClcA proteins reveal five distinct clades of ClcA proteins which were named ClcA-1, ClcA-2, ClcA-3, ClcA-4, and ClcA-5 (see Supplementary Figure 9). *clc*A-1 and *clc*A-3 are located in the same area of the genome in *A. yilgarnensis, A. prosperus*, and *A. aeolianus.* Evidence of the loss of *clcA-3* in *A. yilgarnensis* is evident as genomic synteny is conserved between these genomes (see Figure 6A). Both *clc*A-1 and *clc*A-3 are hypothesized to have been inherited via HGT, as phylogenetic analysis reveals *clc*A-1 clustering closely to ClcA proteins from *Acidithiobacillaceae* and *clc*A-3 clustering with *Sneathiella* spp., *Symmachiella* spp*., Desulfobacteraceae* spp., and *Thiomicrospira* spp. The genes surrounding *clc*A-1 *and clc*A-3 may also be involved in both acid and osmotic tolerance; *pst*ACS (proton buffering), *ygg*TUS (osmotic stress signaling), *pro*H (associated with the production of proline as a compatible solute), and *pil*U and *pil*T (involved in cell adhesion, colonization, biofilm maturation and twitching) (Ito et al., 2009; Chen et al., 2015). *pil*G was identified to be upregulated in *Acidihalobacter* during increasing osmotic stress, thus these proteins may also play a role in osmotic tolerance, although no direct evidence is available of this mechanism in other bacteria (Dopson et al., 2016). This area of the genome may be important to acidic and osmotic tolerance in the *Acidihalobacter*, given that many of the genes in the same context are possibly involved in both osmotic and acidic tolerance. *A. ferrooxydans* is the only *Acidihalobacter* that had a second copy of the *clc*A-1 and is hypothesized to have been acquired via gene duplication of the original *clc*A*-*1 gene and has undergone further diversification. The second copy of *clc*A-1 in *A. ferrooxydans* is directly upstream of the hypothesized original *clc*A-1 gene and clusters closely with the original *clc*A-1 proteins (Figure 6B). *clc*A-2 was only identified in *A. ferrooxydans* and was in an area of the genome with no synteny between any other *Acidihalobacter* genomes (Figure 6A). Phylogenetic analysis indicates the *clc*A-2 protein also clustered closely with ClcA proteins in *Acidithiobacillaceae*, and is hypothesized to have been acquired via HGT from said Acidithiobacillaceae ClcA-4 clusters closely to ClcA proteins from other Chromatiales ClcA proteins, and is in a conserved region of the genome across all the *Acidihalobacter* genomes (see Supplementary Figure 10), thus we suspect that *clc*A-4 was acquired via vertical descent, and subsequently lost in *H. neapolitanus*. ClcA-5 was the only ClcA protein found in the outgroup *H. neapolitanus c2* genome, however, is hypothesized to not be orthologous to any of the ClcA proteins found in the *Acidihalobacter* genomes as it was not phylogenetically related to any of the *Acidihalobacter* ClcA proteins. We hypothesize that the multiple copies of *clc*A in *Acidihalobacter* may point to its usefulness in the acidic response of *Acidihalobacter*. The abundance of chloride ions in *Acidihalobacter* environments could possibly allow for the ClcA mechanism of exporting H^+^ in exchange for Cl^–^ is an effective and energetically favored mechanism, due to the abundance of chloride in *Acidihalobacter* environment. Although this mechanism is found in other acidophiles, it may be more useful for a haloacidophile like *Acidihalobacter* as it is adapted to a high chloride environment.

### Acid Tolerance Genes Absent in the *Acidihalobacter* Genus

Multiple genes that are hypothesized to be involved in acid tolerance were not identified in the *Acidihalobacter* genomes (the complete list of genes queried against *Acidihalobacter* can be found in the Supplementary Table 2). *Acidihalobacter* lacked the lysine, ornithine and agmatine decarboxylase systems. Regulatory genes involved in the glutamate decarboxylase system were absent, including *yba*S, *gad*X, *gad*W, and *gad*E. Alternative regulatory genes could be responsible for the glutamate decarboxylase system in *Acidihalobacter*, and further experimental data may shed light on such genes. Multiple hopanoid biosynthesis genes that produce alternative hopanoid proteins were either absent in the *Acidihalobacter* genomes, or lacked essential intermediate genes. Genes involved in producing general stress proteins were not covered in this study as they are not specialized acid tolerance cellular mechanisms.

### Model of *Acidihalobacter* Acid Resistance

**FIGURE 7:**
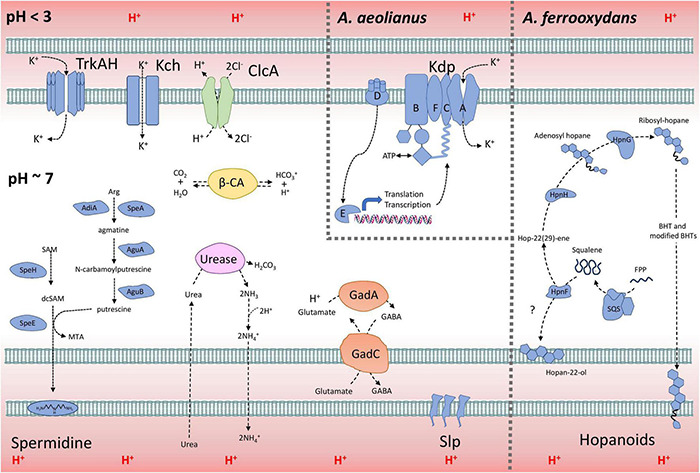
The model of acid resistance in the *Acidihalobacter* spp. First line of defense mechanisms are displayed in blue and second line of defense mechanisms are displayed in other colors (green = *Clc*A; yellow = β-CA; purple = urease system; and orange = Gad system).

A model explaining the acid resistance mechanisms identified in *Acidihalobacter* is proposed in Figure 7. We propose the major mechanisms aiding in the acid resistance of the *Acidihalobacter* spp. to be a combination of potassium transporters, multiple cytoplasmic buffering systems and cellular membrane alterations. The potassium transporters including TrkAH and Kch are likely involved in generating a reversed membrane potential, repelling protons from entering the cell in *Acidihalobacter* spp. *A. aeolianus* has the additional Kdp potassium transport system, possibly aiding it in maintaining a reversed membrane potential when exposed environments low in potassium ions. *Acidihalobacter* has multiple cytoplasmic buffering systems including the glutamate decarboxylase system, the urease system, and carbonic anhydrase. These buffering systems likely contribute to removing any protons that have leaked through the membrane and thus aid in maintaining a circumneutral intracellular pH. The urease system is reported as a strong buffering system, allowing *H. pylori* to survive in the acidity of the stomach. The effectiveness of this system in *Acidihalobacter* is unknown, however, as only *A. ferrooxydans* has a urea transport system. The glutamate decarboxylase system is another well-known buffering system used by enteric bacteria that have transient exposure to the acidic conditions of the stomach. It is evident in the study that the glutamate decarboxylase system is present in *Acidihalobacter* spp. and was likely acquired from acidophilic bacteria. Interestingly, glutamate is also commonly accumulated as an osmoprotectant in halophilic bacteria (Dinnbier et al., 1988; Csonka et al., 1994; Kang and Hwang, 2018), therefore the import of glutamate by the glutamate decarboxylase system may also aid during osmotic stress. Membrane alterations also likely play a role in the acid resistance of *Acidihalobacter* spp. including Slp lipoprotein, spermidine and BHT production in *A. ferrooxydans.* Multiple Slp lipoproteins identified in *Acidihalobacter* likely provide resistance to organic acids, that are detrimental to many chemolithotrophic acidophiles, including *Acidihalobacter.* This area of the genome has also uncovered many genes involved in outer membrane proteins, that may be important to assess for their function in *Acidihalobacter* response to acidic and osmotic stress. BHTs produced in *A. ferrooxydans* likely aid in its resistance to acidic conditions as hopanoids have been identified in acid mine drainage and other acidic environments (Jones et al., 2012). Although the mechanism of spermidine in acid tolerance is not fully understood, *Acidihalobacter* spp. have the genetic potential to produce spermidine, and may aid in its acid tolerance. Multiple ClcA proteins identified in *Acidihalobacter* likely exchange chloride for the removal of protons, which may be especially effective in *Acidihalobacter* as opposed to other acidophiles sensitive to chloride ions.

### Phylogenetic Distribution of Acid Resistance Genes and Their Inferred Evolutionary Trajectories

**FIGURE 8:**
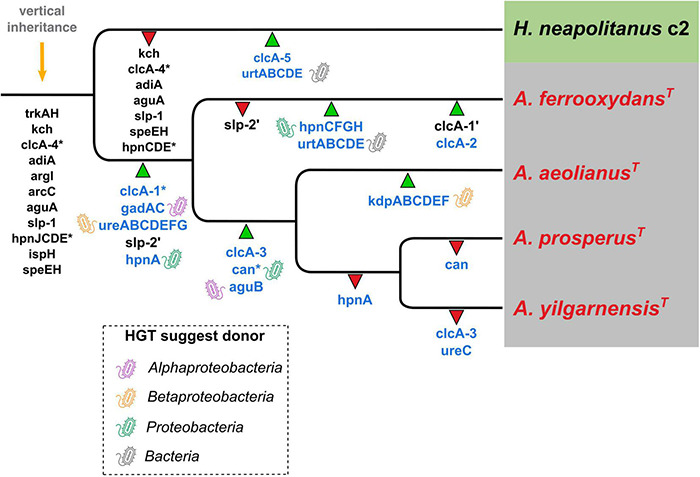
Inferred phylogenetic distribution of acid resistance genes. Green triangles represent gene gain events (the ’ in *slp* and *clc*A genes represent a duplication event) and red triangles represent gene loss events. Gene names in blue indicate hypothesized HGT events (* marks the uncertain origin for that gene) and the bacteria-icon represents the putative origin of those events. Orange arrow = predicted genes inherited by vertical descent.

The hypothesized evolutionary trajectory leading to the *Acidihalobacter* genus becoming acidophilic from an inferred neutrophilic ancestor is summarized in Figure 8, displaying gene gain and gene loss events and gene inheritance through vertical descent. Many genes involved in acid resistance, were hypothesized to be in the last inferred common ancestor including the potassium transporters (*trk*AH, *kch*) and membrane alterations (*hpn*JCDE, *isp*H and *spe*EH). It is evident that the accumulation of potassium via potassium pumps is vital when responding to both osmotic and acidic stress. In this study we hypothesize the TrkAH low affinity potassium transporter to have been acquired via vertical descent from a common halophilic ancestor. Although the Kch proteins have Best-BlastP hits to many Kch proteins from the Acidithiobacillaceae family, phylogenetic analysis reveals the *Acidihalobacter* spp. Kch proteins cluster closely with Kch proteins from organisms of the Chromatiales class. Thus, we inferred the Kch protein to have also been inherited via vertical descent, and subsequently lost in the outgroup. The Kdp high affinity potassium transport system was hypothesized to have been acquired in the *A. aeolianus* genome via HGT likely from Betaproteobacteria, according to the phylogenetic tree (Supplementary Figure 3B). *A. ferrooxydans* BHT synthesis genes were likely obtained in the genome via both vertical descent and HGT. HpnCDE are involved in squalene synthesis, an early intermediate product of BHT production. These protein sequences were phylogenetically related to the same proteins in other Chromatiales species, indicating they were likely vertically inherited. Squalene is known to be associated with halophilic archaea’s membranes, integrating with cellular lipids, helping them to pack closer together, thus is supports HpnCDE likely being inherited from a halophilic common ancestor (Gilmore et al., 2013). Hopanoids are also associated with saline stress, however, the remainder of the BHT synthesis genes (*hpn*FGH) had best-BlastP hits to non-Chromatiales proteins and due to the lack of these genes in the other *Acidihalobacter* genomes, as well as the outgroup, we hypothesize these genes were gained via HGT in the *A. ferrooxydans* genome. All the typical “first line of defense” mechanisms described in extreme acidophiles, in the case of *Acidihalobacter*, seem to originate from a neutrophilic-halophilic ancestry. When it comes to second line of defense mechanisms, it is clear in this study that *Acidihalobacter* has likely gained two cytoplasmic buffering systems through HGT. The glutamate decarboxylase system (*gad*AC) was acquired via HGT into the *Acidihalobacter* genome from a possible acidophilic microbe. Urease (*ure*ABCDEFG) genes were also acquired into the *Acidihalobacter* genome via HGT, but we hypothesize from a non-acidophilic counterpart. Although not acquired from an acidophile, the urease system is known to be a strong buffering system for non-acidophilic enteric bacteria such as *H. pylori*, thus may be able to contribute to acid tolerance in *Acidihalobacter*. Urease was identified in the extreme acidophile *Ferrovum* sp. JA12, and it was also noted in the study that urease has not been identified in any other iron oxidizing acidophiles (Ullrich et al., 2016b). As mentioned previously, it is unclear how effective the urease system is in *A. yilgarnensis, A. prosperus*, and *A. aeolianus*, as no urea transporter was identified in these genomes. ClcA proteins in the *Acidihalobacter* genomes were hypothesized to have independent evolutionary histories. *clc*A-1 was likely acquired via HGT in the *Acidihalobacter* genomes from an acidophilic counterpart and underwent a duplication event in *A. ferrooxydans.* clcA-2 we hypothesize was also acquired via HGT in *A. ferrooxydans* independently. *clc*A-3 was likely acquired via HGT, however, from a non-acidophilic counterpart. *clc*A-4 was the only ClcA protein to have been vertically inherited and subsequently lost in the outgroup as it is phylogenetically related to other ClcA proteins from Chromatiales species. Although ClcA proteins are recognized in the acid stress responses of acidophiles, they have also been identified in some halophilic bacteria, although the function of ClcA proteins in halophiles has not been discussed (John et al., 2019). The evolutionary origin of carbonic anhydrase remains unclear. *Acidihalobacter* has been identified in shared environments with acidophilic microorganisms that we hypothesize had HGT events between species namely members of the Acidithiobacillaceae family (Norris et al., 2020; Abramov et al., 2021). Both *Acidihalobacter* and Acidithiobacillaceae species have been isolated from the acidified sulfide-rich seawater sites from the islands of Vulcano and Milos (Simmons and Norris, 2002; Norris et al., 2020). Other environments that have identified *Acidihalobacter* and other acidophiles inhabiting the same ecological niche include arid soils in the Atacama desert Chile (Neilson et al., 2012) and acidic hypersaline river sediments of Western Australia (Lu et al., 2016). The evidence of professional acidophiles and *Acidihalobacter* supports environmental scenarios for HGT events to have occurred between these bacteria. These environmental niches hypothetically provide the ideal conditions for acidophiles and halophiles to live in close proximity and also apply the selection pressure for existing halophiles and acidophiles alike to share genetic capabilities. We hypothesize *Acidihalobacter* is an example of a halophile gaining acid tolerance mechanisms, however, the opposite case may still be discovered in other bacteria. The cellular mechanisms for microorganisms to thrive in saline and acidophilic conditions independently is well established. Understanding what combination of these cellular mechanisms *Acidihalobacter* has, that allows it to cope in both extreme acidity and saline conditions will give insight into the unique capabilities of haloacidophiles. The current understanding of *Acidihalobacter* as a halophile, is that it utilizes potassium pumps, periplasmic glucans and multiple compatible solutes to regulate its internal osmotic pressure to its saline environment. It is proposed by Khaleque et al. (2019) that initially potassium accumulation and the production of periplasmic glucans are the primary mechanisms used to respond to initial saline stress, and as salinity increases, the accumulation and production of compatible solutes would take priority. Identifying the genetic capabilities *Acidihalobacter* spp. possess that allows the genus to thrive in acidic conditions is vital to understand its evolution as a polyextremophile.

It is evident that *Acidihalobacter* is a genus rooted in a class of bacteria that are mostly halophilic/halotolerant neutrophiles and alkaliphiles. *Acidihalobacter* is also the only genus in the Chromatiales order that has been found to be acidophilic. We hypothesize that the ancestor of *Acidihalobacter* was a halophile that subsequently gained the genetic capability to thrive in acidic conditions. This research has identified some of these hypothesized genes involved in acid tolerance and uncovered evidence of HGT events that may have been involved in *Acidihalobacter* becoming acidophilic. The main mechanisms identified that likely are involved in acid tolerance in *Acidihalobacter* spp. include the potassium transporters TrkAH, Kch, and Kdp, membrane-associated alterations Slp, spermidine and BHTs, cytoplasmic buffering mechanisms, glutamate decarboxylase, urease and carbonic anhydrase, and the antiporters ClcA’s. This study can inform further experimentation studying the acid tolerance of *Acidihalobacter.*

## Data Availability Statement

Publicly available datasets were analyzed in this study. This data can be found here: NCBI GCF_000754095.2, GCF_001753245.1, GCF_001753165.1 GCF_001975725.1, and GCF_000024765.1.

## Author Contributions

DH and EW conceived the study. KB carried out the research. All authors contributed to data collection, analysis, and manuscript preparation and read and approved the final manuscript.

## Conflict of Interest

The authors declare that the research was conducted in the absence of any commercial or financial relationships that could be construed as a potential conflict of interest.

## Publisher’s Note

All claims expressed in this article are solely those of the authors and do not necessarily represent those of their affiliated organizations, or those of the publisher, the editors and the reviewers. Any product that may be evaluated in this article, or claim that may be made by its manufacturer, is not guaranteed or endorsed by the publisher.

## Footnotes

1
https://itol.embl.de/
