# Supplementary material for: Prediction and Inferred Evolution of Acid Tolerance Genes in the Biotechnologically Important Acidihalobacter Genus
Source: Front Microbiol. 2022 Apr 18;13:848410. doi: 10.3389/fmicb.2022.848410 (PMC9062700; doi:10.3389/fmicb.2022.848410)

Supplementary Material

Prediction and Inferred Evolution of Acid Tolerance Genes in the Biotechnologically Important *Acidihalobacter* Genus

***Katelyn Boase[1], *Carolina González[2], Eva Vergara[2,3], Gonzalo Neira[2], #David Holmes[2,3] and #Elizabeth Watkin[1]**

[1]Curtin Medical School, Curtin University, Perth, WA, Australia

[2]Center for Bioinformatics and Genome Biology, Centro Ciencia & Vida, Fundación Ciencia & Vida, Santiago, Chile

[3]Facultad de Medicina y Ciencias, Universidad San Sebastián, Santiago, Chile

*Co-first authors

# Co-Corresponding authors

Supplementary Table S1: Representative Chromatiales Proteomes and Associated Details used for the Phylogenetic Tree Including all *Acidihalobacter* proteomes.

Genus and species	Intraspecific name	DSM Number	GenBank Assembly ID	pH opt (range)	NaCl opt (range) %w/v	Growth without NaCl?	Halophile?	Acidophile <4 Alkaliphile >9
Acidihalobacter aeolianus	V6	DSM 14174	GCA_001753165.1	1.8 (1.5-3)	2.34 (0.35-7.5)	No	Slight halophile	Acidophile
Acidihalobacter ferrooxydans	V8	DSM 14175	GCA_001975725.1	1.8 (1-3)	2.34 (0.35-5)	No	Slight halophile	Acidophile
Acidihalobacter prosperus	DSM 5130	DSM 5130	GCA_000754095.2	2 (1-4.5)	1.2 (0.41-5.8)	No	Slight halophile	Acidophile
Acidihalobacter yilgarnenesis	F5	DSM 105917	GCA_001753245.1	2.5 (2-4)	2.34 (0.03-7.5)	No	Slight halophile	Acidophile
Alkalilimnicola ehrlichii	MLHE-1	DSM 17681	GCA_000014785.1	9.3	3 (1.5-19)	No	Slight halophile	Alkaliphile
Allochromatium vinosum	DSM 180	DSM 180	GCA_000025485.1	(6.5 - 7.6)	0	Yes	Non-halotolerant	Neutrophile
Allochromatium warmingii	DSM 173	DSM 173	GCA_900107145.1	7.0–7.3 (6.5–7.6)	0	Yes	Non-halotolerant	Neutrophile
Aquisalimonas asiatica	CGMCC 1.6291	DSM 18102	GCA_900110585.1	7.5-8.5 (6-10.8)	7-10 (1-20)	No	Moderate halophile	Neutrophile
Ectothiorhodosinus mongolicus	M9	DSM 15479	GCA_900156225.1	8.3-9.1 (7.6-10.1)	1-7 (0.5-9)	No	Moderate halophile	Alkaliphile
Ectothiorhodospira magna	B7-7	DSM 22250	GCA_900110965.1	9-10 (8-11)	0.5-1.5 (0-8)	No	Moderate halophile	Alkaliphile

Ectothiorhodospira marina	DSM 241	DSM 241	GCA_900109495.1	7.5-8.5	2-6 (0.5-10)	No	Moderate halophile	Neutrophile
Ectothiorhodospira mobilis	DSM 4180	DSM 4180	GCA_900114895.1	7.6-8	2-3 (1-5)	No	Slight halophile	Neutrophile
Granulosicoccus antarcticus	IMCC3135	DSM 24912	GCA_002215215.1	7	2 (0.5-5)	No	Slight halophile	Neutrophile
Halorhodospira halophila	SL1	DSM 244	GCA_000015585.1	8.5-9	11-12 (3-30)	No	Moderate halophile	Alkaliphile
Halothiobacillus neapolitanus	c2	DSM 15147	GCA_000024765.1	6.5-6.9 (4.5-8.5)	ND (0-5)	Yes	Slightly halotolerant	Neutrophile
Imhoffiella purpurea	AK35		GCA_000585215.1	7.5-8.5 (7-8.5)	1-2 (0-4)	No	Slight halophile	Neutrophile
Marichromatium purpuratum	984	DSM 1591	GCA_000224005.3	7.2-7.6	5 (2-7)	No	Moderate halophile	Neutrophile
Nitrococcus mobilis	Nb-231		GCA_000153205.1	7.5-8	70-100% seawater	No	Moderate halophile	Neutrophile
Nitrosococcus halophilus	Nc 4		GCA_000024725.1	7.6-8	4.1	No	Slight halophile	Neutrophile
Nitrosococcus oceani	ATCC 19707		GCA_000012805.1	7.6-8	2.9	No	Slight halophile	Neutrophile
Nitrosococcus watsonii	C-113		GCA_000143085.1	7.6-8	9.35	No	Moderate halophile	Neutrophile
Oceanococcus atlanticus	22II-S10r2		GCA_002088235.1	7-8 (6-10)	3 (0.5-7)	No	Slight halophile	Neutrophile
Rheinheimera salexigens	KH87	DSM 100018	GCA_001752395.1	6.5-7.6 (6-8)	1-3 (0.5-5)	Yes	Slightly halotolerant	Neutrophile
Spiribacter salinus	M19-40		GCA_000319575.2	7.5-8 (6-9)	15 (10-25)	No	Moderate halophile	Neutrophile

Thioalkalivibrio denitrificans	ALJD	DSM 13742	GCA_002000365.1	9.6-10 (8-10.5)	3.5 (1.17-23)	No	Slight halophile	Alkaliphile
Thioalkalivibrio halophilus	HL17	DSM 15791	GCA_001995255.1	8-9 (7.3-9.8)	11.7 (1.7-29.22)	No	Moderate halophile	Alkaliphile
Thioalkalivibrio nitratireducens	DSM 14787	DSM 14787	GCA_000321415.2	9.5 10 (8-10.5)	2.34-2.92 (1.17-8.77)	No	Slight halophile	Alkaliphile
Thioalkalivibrio paradoxus	ARh1	DSM 13541	GCA_000227685.3	9.8-10.0 (9.5-10.2)	NA	No	Moderate halophile	Alkaliphile
Thioalkalivibrio sulfidiphilus	HL-EbGr7		GCA_000021985.1	10 (8-10.5)	2.34 (1.17-8.77)	No	Slight halophile	Alkaliphile
Thioalkalivibrio versutus	D301		GCA_001020955.1	10-10.2 (7.5-10.65)	Upper salt limit 23.38%	No	Moderate halophile	Alkaliphile
Thiocapsa marina	5811	DSM 5653	GCA_000223985.2	7.5	1-2 (0-8)	Yes	Slightly halotolerant	Neutrophile
Thiocystis violascens	DSM 198	DSM 198	GCA_000227745.3	7-7.3	0	Yes	Non-halotolerant	Neutrophile
Thioflavicoccus mobilis	8321		GCA_000327045.1	7.2-7.4	2	No	Slight halophile	Neutrophile
Thiohalomonas denitrificans	HLD2	DSM 15841	GCA_900102855.1	7.3-8.2 (6.5-8.2)	8.77-11.7 (5.8-17.5)	No	Moderate halophile	Neutrophile
Thiohalospira halophila	DSM 15071	DSM 15071	GCA_900112605.1	7.3-7.8 (6.5-8.2)	14.61-17.5 (11.7-29.22)	No	Moderate halophile	Neutrophile

Thiorhodococcus drewsii	AZ1	DSM 15006	GCA_000224065.2	6.5-6.7	2.4-2.6 (0-8)	No	Slight halophile	Neutrophile
Wenzhouxiangella marina	KCTC 42284	DSM 103414	GCA_001187785.1	7-8 (6-9)	3-5 (max 13)	No	Slight halophile	Neutrophile
Woeseia oceani	XK5		GCA_001677435.1	7-8 (6-9)	1-3 (0.5-8)	No	Slight halophile	Neutrophile
Pararheinheimera texasensis	DSM 17496	DSM 17496	GCA_000711985.1	7.75 (6.5-9)	0-1	Yes	Non-halotolerant	Neutrophile
Rheinheimera perlucida	DSM 18276	DSM 18276	GCA_000382165.1	7 (5.7-10)	1-3 (0-8)	Yes	Slightly halotolerant	Neutrophile
Rheinheimera nanhaiensis	DSM 22401	DSM 22402	GCA_000296695.1	7.5-8.5 (5.5-10)	0.5-2.5 (0-8)	Yes	Slightly halotolerant	Neutrophile
Rheinheimera baltica	DSM 14885	DSM 14885	GCA_000425345.1	7 (5.7-10)	1-3 (0-6)	Yes	Slightly halotolerant	Neutrophile
Halofilum ochraceum			GCA_001614315.2	7.5-8 (6.5-9.5)	8-10 (2-20)	No	Moderate halophile	Neutrophile
Arhodomonas aquaeolei	DSM 8974	DSM 8974	GCA_000374645.1	6.5-7.5 (6-8)	15 (6-20)	No	Moderate halophile	Neutrophile
Lamprocystis purpurea	DSM 4197	DSM 4197	GCA_000379525.1	7-7.3	NA	NA	NA	Neutrophile
Thioalkalivibrio thiocyanoxidans	ARh2	DSM 13543	GCA_000385215.1	10	(1.75-5.8)	No	Slight halophile	Alkaliphile
Thioalkalivibrio thiocyanodenitrificans	ARhD 1	DSM 16954	GCA_000378965.1	9.6-10	(1.75-10.5)	No	Slight halophile	Alkaliphile
Thiorhodospira sibirica	ATCC 700588		GCF_000227725.2	9	(0-6)	Yes	Slightly halotolerant	Alkaliphile
Ectothiorhodospira haloalkaliphila	ATCC 51935		GCA_000633935.1	8.5-10	5 (2.5-15)	No	Moderate halophile	Alkaliphile

Supplementary Table S2. Genes that were queried in *Acidihalobacter* that are hypothesized to be involved in acid tolerance. ND indicates genes not detected.

	A. yilgarnensis	*A. prosperus*	*A. aeolianus*	*A. ferrooxydans*	*H. neapolitanus* c2
**Potassium transport**					
kdpA	ND	ND	AOV15972.1	ND	ND
kdpB	ND	ND	AOV15971.1	ND	ND
kdpC	ND	ND	AOV15970.1	ND	ND
kdpD	ND	ND	AOV15969.1	ND	ND
trkA	AOU96728.1	OBS08269.1	AOV18191.1	APZ44371.1	ACX96897.1
trkH	AOU96729.1	OBS08268.1, OBS08267.1	AOV18190.1, AOV18189.1	APZ44370.1, APZ44767.1	ACX96896.1
kch	AOU99651.1	OBS10739.1	AOV18503.1	APZ44536.1	ND
nhaA	AOU96684.1	OBS08340.1	AOV18240.1	APZ44487.1	ND
**Na/H and K/H antiporters**					
AFE_0791 Na/H exchanger	AOU98934.1	OBS10243.1	AOV17801.1	ND	ND
AFE_1968 K/H antiporter	AOU98934.1	OBS10243.1	AOV17801.1	APZ44223.1	ACX96798.1
clcA -1	AOU99460.1	OBS08149.1,	AOV18657.1	APZ43554.1, APZ44545.1, APZ44679.1	ND
clcA-2	AOU99515.1	OBS08582.1	AOV17735.1	APZ44468.1	ND
clcA-3	ND	OBS08154.1	AOV18114.1	ND	ND
**Glutamate dependant acid resistance**					
GadA	AOU97331.1	OBS08746.1	AOV18434.1	APZ43583.1	ND
gadC	AOU99537.1	OBS08747.1	AOV16509.1	APZ43581.1	ND
gadX	ND	ND	ND	ND	ACX94995.1
gadW	ND	ND	ND	ND	ACX94990.1, ACX94995.1
ybaS	ND	ND	ND	ND	ND
gadE	ND	ND	ND	ND	ND
**Arginine dependant acid resistance**					
adiA	AOU99171.1	OBS09300.1	AOV15941.1	APZ44349.1	ND
adiC	AOU99537.1, AOU97404.1	OBS08831	AOV18445.1	APZ43581.1, APZ42099.1	ND
**Deaminase systems**					
argI	AOU97246.1	OBS08618.1	AOV17707.1	APZ42265.1	ACX96179.1
arcA	ND	ND	ND	ND	ND
arcC	ND	OBS08297.1	ND	ND	ND

aguA	AOU98013.1	OBS10878.1	AOV17048.1	APZ42501.1	ND
aguB	AOU97411.1	OBS08838.1	AOV16576.1	ND	ND
**Lysine**					
cadA	ND	ND	ND	ND	ACX95924.1
cadB	AOU99537.1	ND	ND	APZ43581.1	ND
cadC	ND	ND	ND	ND	ND
cadR	ND	ND	ND	ND	ND
**Ornithine**					
speC	ND	ND	ND	ND	ACX95924.1
speF	ND	ND	ND	ND	ACX95924.1
potE	ND	ND	ND	ND	ND
**Acid fitness island**					
yhiD	ND	ND	ND	ND	ND
slp-1	AOU99100.1	OBS09214.1	AOV16013.1	APZ44451.1	ND
slp-2	AOU99791.1	OBS09215.1	AOV18359.1	ND	ND
yhiF	ND	ND	ND	ND	ND
hdeD	ND	ND	ND	ND	ND
**Urea breakdown**					
ureA	AOU99167.1	OBS09296.1	AOV18345.1	APZ41769.1	ND
ureB	AOU99165.1	OBS09294.1	AOV15946.1	APZ41771.1	ND
ureC		OBS09293.1	AOV15947.1	APZ41772.1	ND
ureD	AOU99168.1	OBS09297.1	AOV15944.1	APZ44328.1	ND
ureE	AOU99797.1	OBS09247.1	AOV15989.1	APZ44456.1	ND
ureF	AOU99798.1	OBS09246.1	AOV15990.1	APZ41774.1	ND
ureG	AOU99123.1	OBS09245.1	AOV18354.1	APZ41741.1	ND
ureI	ND	ND	ND	ND	ND
urtA	ND	ND	ND	APZ44330.1	ACX96427.1, ACX95939.1
urtB	ND	ND	ND	APZ44759.1	ACX96426.1, ACX95938.1
urtC	ND	ND	ND	APZ44758.1	ACX96425.1, ACX95937.1
urtD	ND	ND	ND	APZ44329.1	ACX96424.1, ACX95936.1
urtE	ND	ND	ND	APZ44757.1	ACX95935.1, ACX96423.1
**Hopanoids**					
hpnF	ND	ND	ND	APZ44550.1	ND
hpnH	ND	ND	ND	APZ42479.1	ND
hpnG	ND	ND	ND	APZ42476.1	ND
hpnO	AOU97247.1	OBS08619.1	AOV17706.1	APZ42266.1	ACX96180.1
hpnA	ND	ND	ND	APZ44549.1	ND

hpnB	ND	OBS08108.1, OBS09043.1	ND	APZ43080.1, APZ43964.1	ND
hpnM	AOU99024.1	OBS09099.1	AOV18637.1	APZ42457.1	ACX95612.1
hpnK	ND	ND	ND	ND	ACX96354.1
hpnJ	ND	ND	ND	APZ43616.1	ND
hpnH	ND	ND	ND	ND	ND
hpnI	ND	ND	ND	ND	ND
hpnL	ND	ND	ND	ND	ND
hpnC	AOU99599.1	OBS10465.1	AOV17210.1	APZ42803.1	ND
hpnD	AOU97624.1	OBS10464.1	AOV17211.1	APZ42452.1	ND
hpnE	AOU99598.1	OBS10463.1	AOV17212.1	APZ42451.1	ND
ispH	AOU99394.1	OBS09541.1	AOV18271.1	APZ42478.1, APZ44101.1	ACX97044.1
sqhC	ND	ND	ND	ND	ND
**Proton consuming reactions**					
can	AOU99136.1	ND	AOV15976.1	ND	ND
**Spermidine**					
speE	AOU99172.1	OBS09301.1	AOV15940.1	APZ44348.1	ND
speH	AOU96886.1	OBS08068.1	AOV18052.1	APZ41841.1	ND
**Phosphate uptake**					
pstA	AOU98388.1, AOU96821.1	OBS09730.1, OBS08155.1	AOV16706.1, AOV18658.1	APZ42710.1, APZ44564.1	ACX96618.1
pstB	AOU99677.1	OBS09729.1	AOV18477.1	APZ44684.1	ACX96617.1
pstC	AOU99678.1, AOU96820.1	OBS09731.1, OBS08156.1	AOV18476.1, AOV18115.1	APZ42709.1, APZ42707.1	ACX96619.1
pstS	AOU98389.1, AOU96819.1	OBS09732.1, OBS08157.1	AOV18475.1, AOV18116.1	APZ44565.1, APZ42708.1	ACX96620.1
lexA	AOU99361.1	OBS09506.1	AOV15739.1	APZ43204.1	ACX95728.1
clpP	AOU97504.1	OBS10306.1	AOV17350.1	APZ43588.1, APZ44367.1	ACX95243.1
clpX	AOU97505.1	OBS10307.1	AOV17349.1	APZ43587.1	ACX95244.1
**Glycosyl transferase**					
AFE_1776 glycosyl tranferase	ND	ND	ND	ND	ACX96357.1
AFE_1357 glycosyl transferase	AOU98645.1	OBS09986.1	AOV17566.1	APZ42832.1	ND

Supplementary Information 1. Genes predicted to be involved in mechanisms of HGT in *Acidihalobacter*.

1) Mobilization protein (APZ41764.1) with a conjugal transfer TraL domain (Zhang S and Meyer R. Mol. Microbiol. 1997 Aug;25(3):509-16) was detected in the same genomic context as ureA in in *A. ferrooxydans* strain V8.

2) There is a truncated integrase C-term domain (TIGR02249, integron integrase) upstream of *hpn*F,G H in *A. ferrooxydans* strainV8. Members of this family of integrases are typically associated with integrons (and super-integrons), which are systems for incorporating and expressing cassettes of laterally transferred DNA. Integron-integrases are present in many natural occurring mobile elements, including transposons and conjugative plasmids (Escudero J. A. et al., Na communications. 2016 Mar 10;7(1):1-2).

3) The recombination-associated gene *him*A (Yang, S. W., and Nash, H. A. (1995). EMBO J. 14, 6292–6300) is found in the same genomic context as *gad*AC in *A. ferrooxydans* strainV8.

4) The recombination-associated gene *mut*S2 (Burby P. E. and Simmons L. A. J. Bacteriol. 2017 Jan 1;199(2): e00682-16) is found in the same genomic context as *gad*AC in *A. prosperus* and *A. aeolianus*.

Supplementary Figure S1. Genomic context of *trk* genes in the *Acidihalobacter* genomes and *H. neapolitanus*. Grey background shows synteny between genomes; Red= involved in acid and osmotic tolerance, blue= other genes.

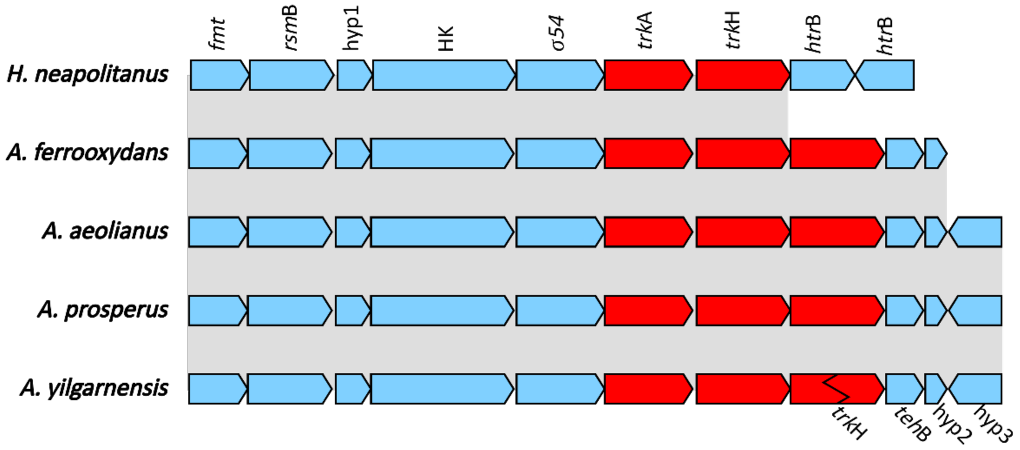

Supplementary Figure S2. Unrooted phylogenetic tree of the predicted *Acidihalobacter* TrkA and TrkH amino acid sequences and their best hits from the NCBI non-redundant database. In red are the *Acidihalobacter* sequences and in green are other members of Chromatiales order.

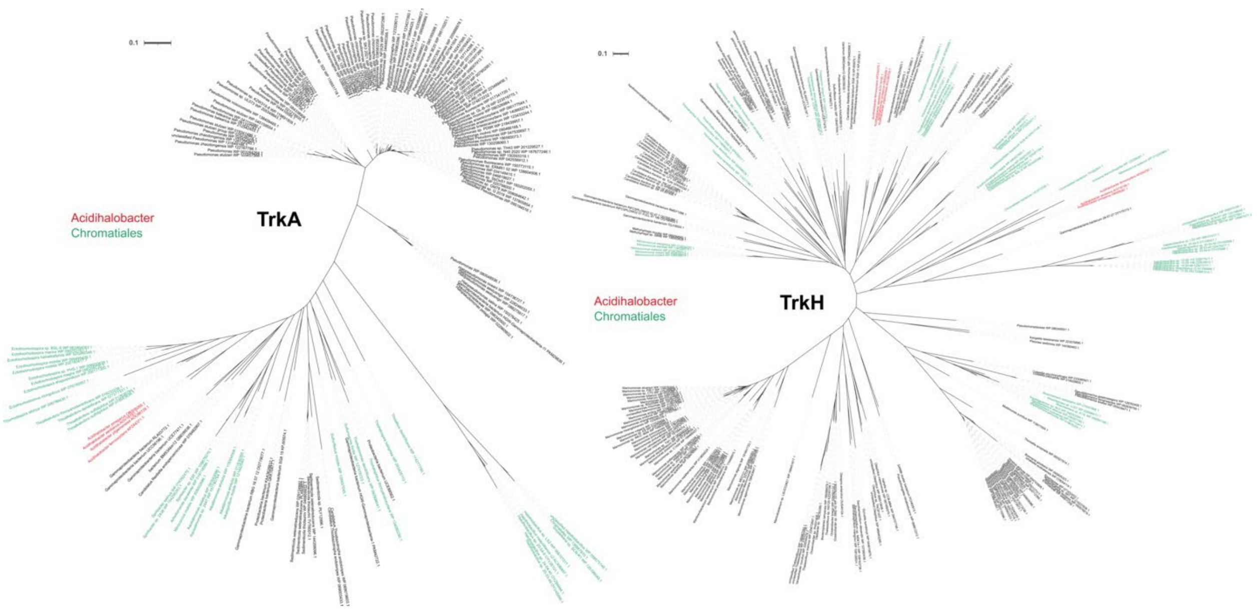

Supplementary Figure S3. A) *kdp* genome context in *A. aeolinaus*. Red= involved in acid and osmotic tolerance, blue= other genes. 1, class I SAM-dependant methyltransferase protein; 2, Glutathione S-transferase N-terminal domain containing protein; 3, sigma-54-dependant Fis family transcriptional regulator and 4, a zinc ribbon domain-containing protein. B) Phylogenetic tree of kdp operon DNA best hits with bootstrap >= 60% in gray bullets at the middle of each branch.

A.

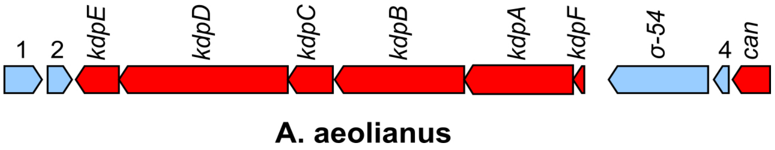

B.

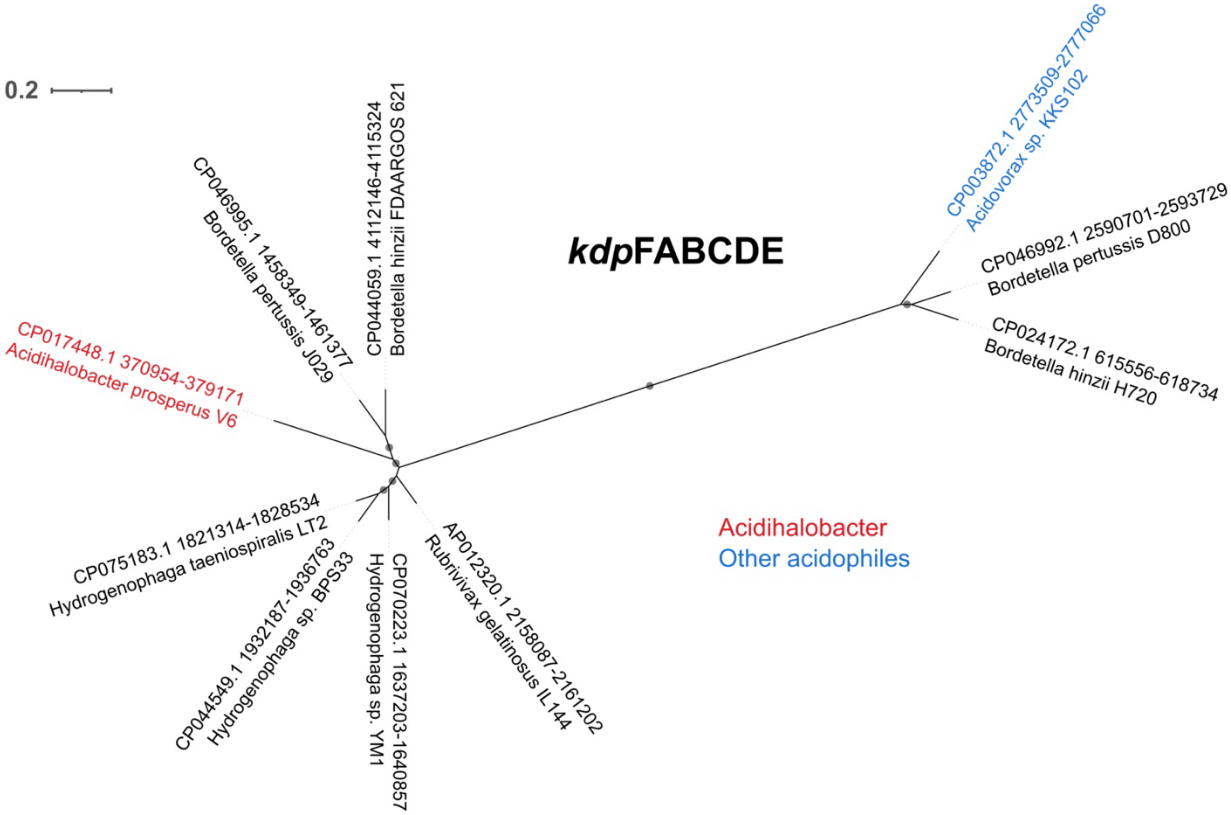

Supplementary Figure S4. Unrooted phylogenetic tree of the predicted *Acidihalobacter* Kch amino acid sequences and their best hits from the NCBI non-redundant database. *Acidihalobacter* Kch proteins are colored red, Chromatiales proteins are colored green and Other acidophiles proteins are colored blue. The time scale bar represents 0.2 amino acid substitution per site.

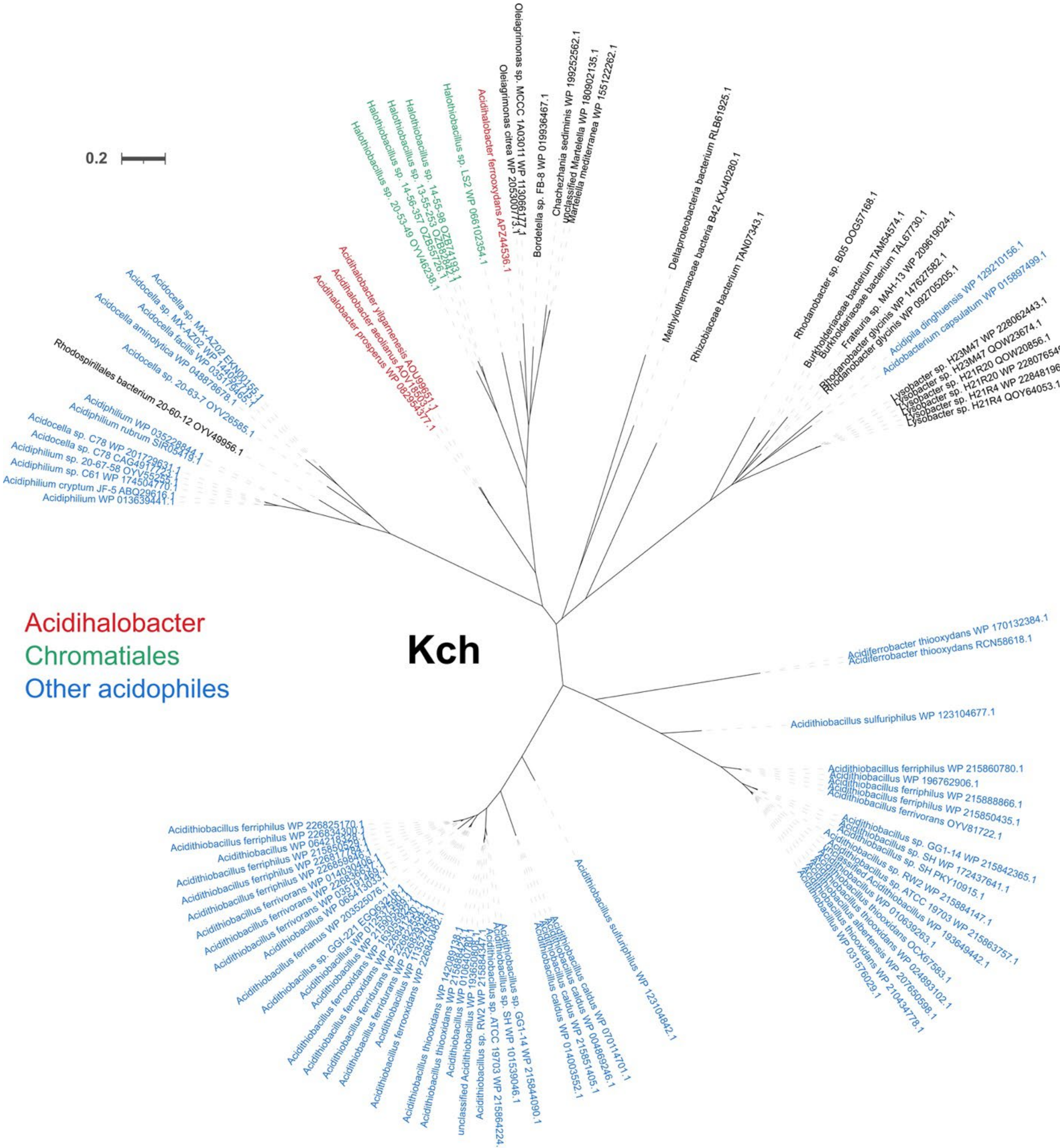

Supplementary Figure S5. Multiple sequence alignment of *Acidihalobacter* genus Slp protein sequences including a WebLogos plot of the slp lipobox.

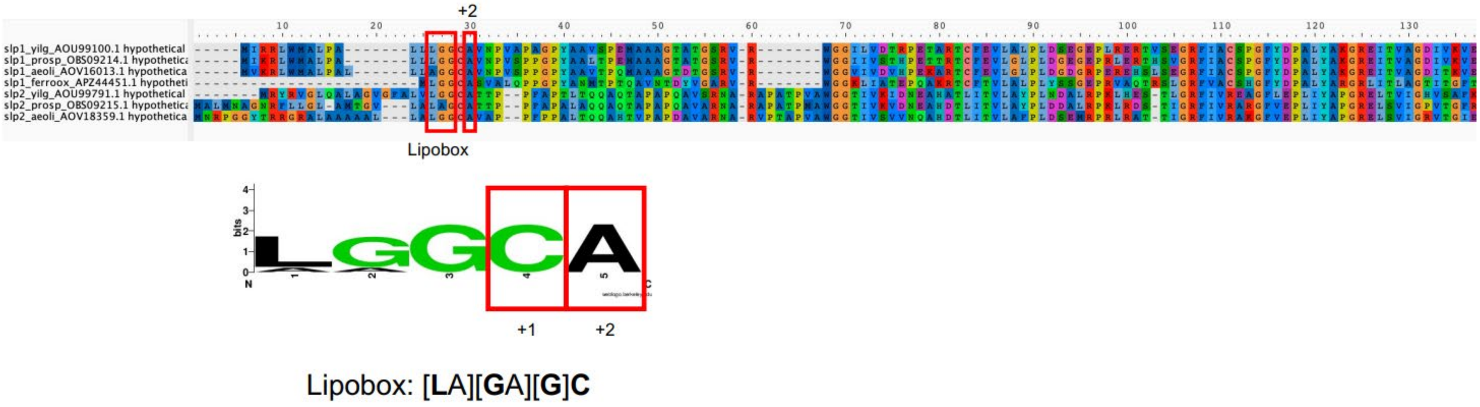

Supplementary Figure S6. Genomic context of *slp* genes in the *Acidihalobacter* genomes and *H. neapolitanus*. Grey background shows synteny between genomes; Red= involved in acid and osmotic tolerance, blue= other genes.

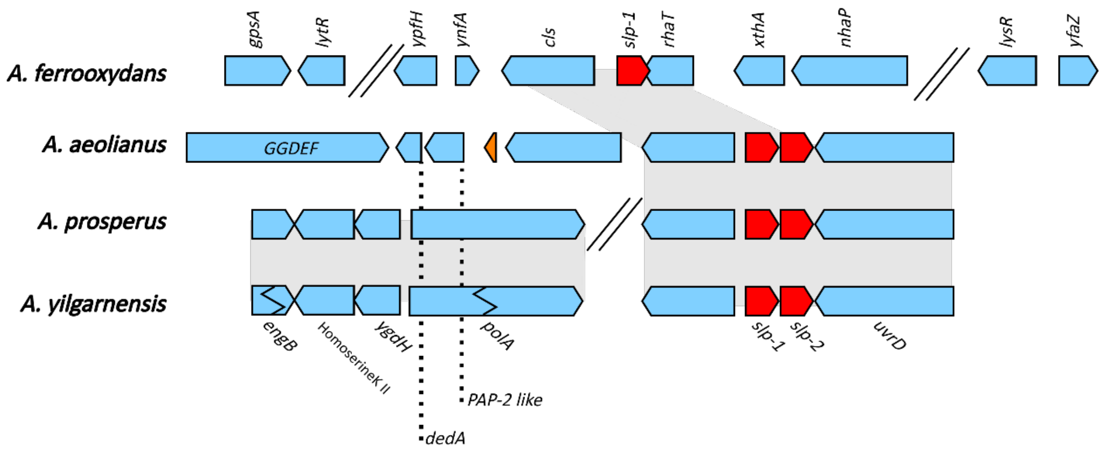

Supplementary Figure S7. Unrooted phylogenetic tree of the predicted *Acidihalobacter* A) GadA and B) GadC amino acid sequences and their best hits from the NCBI non-redundant database. *Acidihalobacter* Gad proteins are colored red, Chromatiales proteins are colored green and other acidophiles proteins are colored blue. The time scale bar represents 0.1 amino acid substitution per site.

A

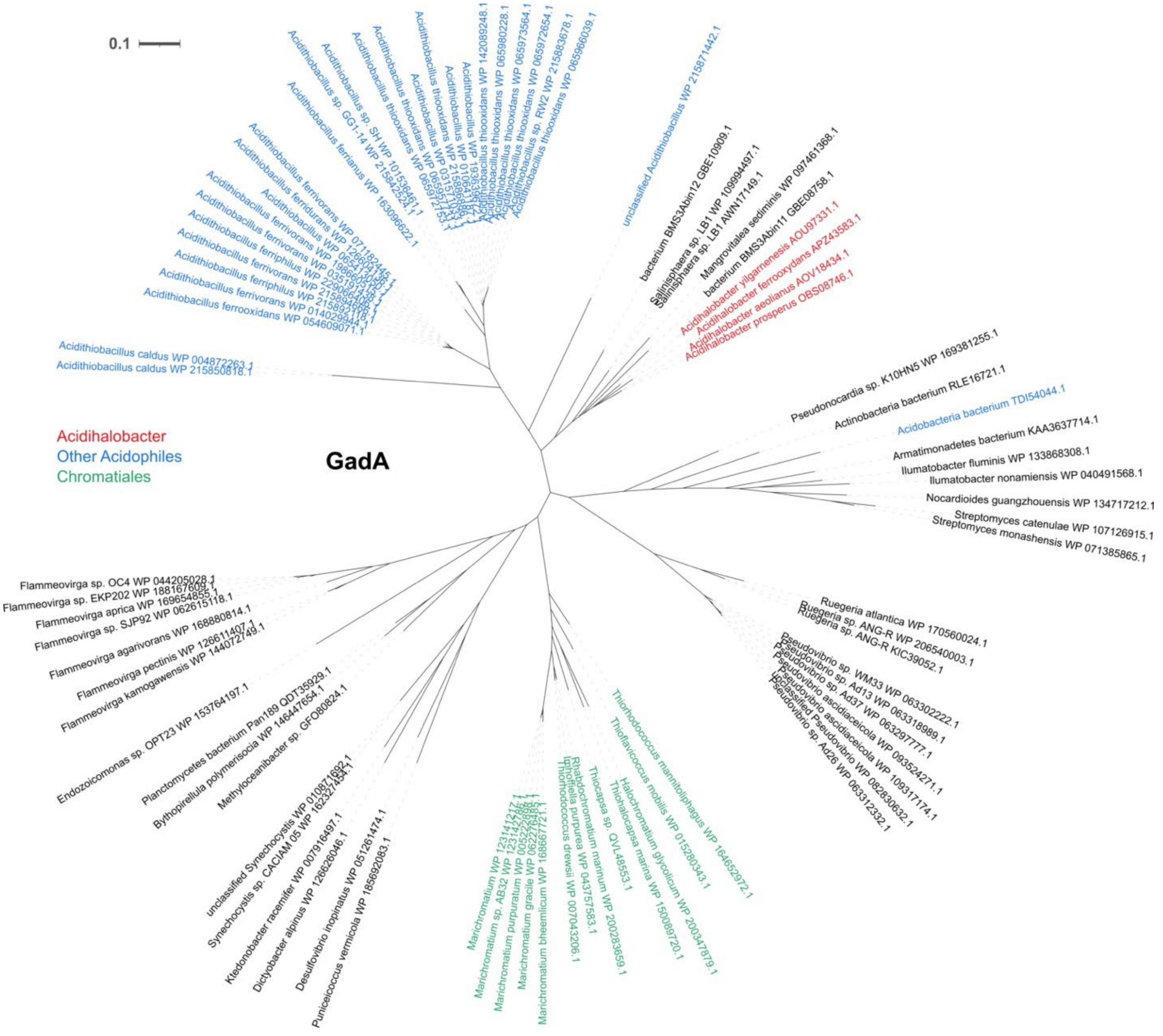

B

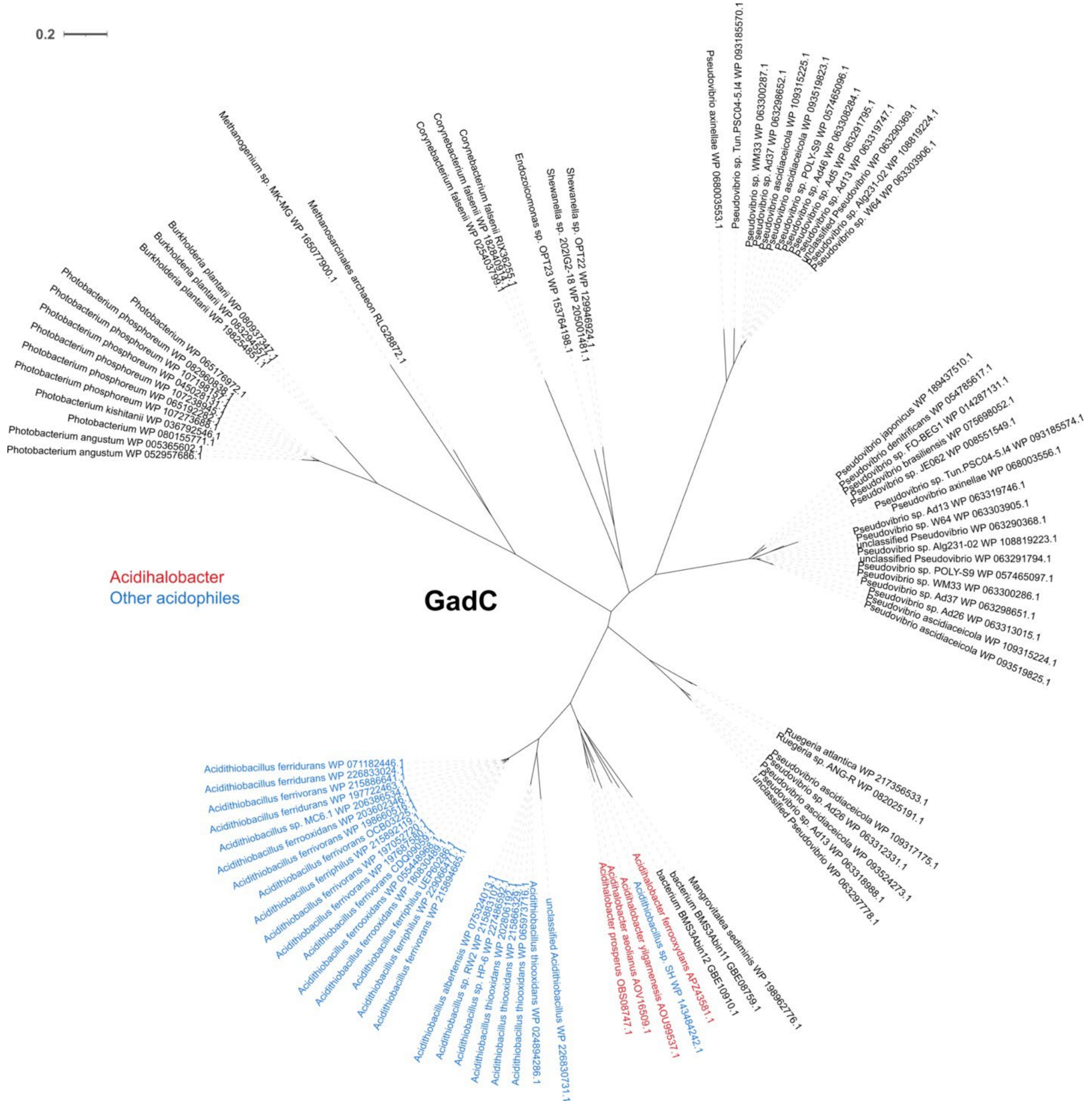

0.2
GadC
Acidihalobacter
Other acidophiles
Pseudovibrio axinellae WP 068003553.1
Pseudovibrio sp. Tun.PSC04-5.I4 WP 093185570.1
Pseudovibrio sp. WM33 WP 063300287.1
Pseudovibrio sp. Ad37 WP 063298652.1
Pseudovibrio ascidiaceicola WP 109315225.1
Pseudovibrio sp. POLY-S9 WP 093519823.1
Pseudovibrio sp. Ad46 WP 057465096.1
Pseudovibrio sp. Ad5 WP 063308284.1
Pseudovibrio sp. Ad13 WP 063291795.1
unclassified Pseudovibrio WP 063319747.1
Pseudovibrio sp. Alg231-02 WP 063290369.1
Pseudovibrio sp. W64 WP 108819224.1
WP 063303906.1
Shewanella sp. OPT22 WP 129946924.1
Shewanella sp. 202IG2-18 WP 205001481.1
Endozoicomonas sp. OPT23 WP 153764198.1
Corynebacterium falsenii RIX36255.1
Corynebacterium falsenii WP 182840914.1
Corynebacterium falsenii WP 025403799.1
Methanosarcinales archaeon RLG28872.1
Methanogenium sp. MK-MG WP 165077900.1
Burkholderia plantarii WP 080937347.1
Burkholderia plantarii WP 083294557.1
Burkholderia plantarii WP 198254851.1
Photobacterium WP 065176972.1
Photobacterium phosphoreum WP 082960938.1
Photobacterium phosphoreum WP 107198136.1
Photobacterium phosphoreum WP 045028131.1
Photobacterium phosphoreum WP 107238947.1
Photobacterium phosphoreum WP 065192682.1
Photobacterium phosphoreum WP 107273688.1
Photobacterium kishitanii WP 036792546.1
Photobacterium WP 080155771.1
Photobacterium angustum WP 005365602.1
Photobacterium angustum WP 052957686.1
Pseudovibrio japonicus WP 189437510.1
Pseudovibrio denitrificans WP 054785617.1
Pseudovibrio sp. FO-BEG1 WP 014287131.1
Pseudovibrio brasiliensis WP 075698052.1
Pseudovibrio sp. JE062 WP 008551549.1
Pseudovibrio sp. Tun.PSC04-5.I4 WP 093185574.1
Pseudovibrio axinellae WP 068003556.1
Pseudovibrio sp. Ad13 WP 063319746.1
Pseudovibrio sp. W64 WP 063303905.1
unclassified Pseudovibrio WP 063290368.1
Pseudovibrio sp. Alg231-02 WP 108819223.1
unclassified Pseudovibrio WP 063291794.1
Pseudovibrio sp. POLY-S9 WP 057465097.1
Pseudovibrio sp. WM33 WP 063300286.1
Pseudovibrio sp. Ad37 WP 063298651.1
Pseudovibrio sp. Ad26 WP 063313015.1
Pseudovibrio ascidiaceicola WP 109315224.1
Pseudovibrio ascidiaceicola WP 093519825.1
Ruegeria atlantica WP 217356533.1
Ruegeria sp. ANG-R WP 082025191.1
Pseudovibrio ascidiaceicola WP 109317175.1
Pseudovibrio sp. Ad26 WP 063312331.1
Pseudovibrio ascidiaceicola WP 093524273.1
Pseudovibrio sp. Ad13 WP 063318988.1
unclassified Pseudovibrio WP 063297778.1
Mangrovitalea sediminis WP 198962776.1
bacterium BMS3Abin11 GBE08759.1
bacterium BMS3Abin12 GBE10910.1
Acidihalobacter ferrooxydans AP724581.1
Acidithiobacillus sp. SH WP 143484242.1
Acidihalobacter yilgarnensis AOU99537.1
Acidihalobacter aeolianus AOV16509.1
Acidihalobacter prosperus OBS08747.1
unclassified Acidithiobacillus WP 226830731.1
Acidithiobacillus thiooxidans WP 024894286.1
Acidithiobacillus thiooxidans WP 065973716.1
Acidithiobacillus thiooxidans WP 215866326.1
Acidithiobacillus thiooxidans WP 202806192.1
Acidithiobacillus sp. HP-6 WP 227146592.1
Acidithiobacillus sp. RW2 WP 215883103.1
Acidithiobacillus albertensis WP 075324013.1
Acidithiobacillus ferridurans WP 071182446.1
Acidithiobacillus ferridurans WP 226833024.1
Acidithiobacillus ferrivorans WP 215886641.1
Acidithiobacillus ferridurans WP 197722463.1
Acidithiobacillus sp. MC6.1 WP 206386534.1
Acidithiobacillus ferrooxidans WP 203602346.1
Acidithiobacillus ferrivorans WP 198660516.1
Acidithiobacillus ferrivorans OCB03225.1
Acidithiobacillus ferriphilus WP 215892113.1
Acidithiobacillus ferrivorans WP 197057220.1
Acidithiobacillus ferrivorans CDQ09008.1
Acidithiobacillus ferrooxidans WP 197687599.1
Acidithiobacillus ferrooxidans WP 055448988.1
Acidithiobacillus ferriphilus UEP60286.1
Acidithiobacillus ferriphilus WP 180804898.1
Acidithiobacillus ferrivorans WP 229986441.1
WP 215894665.1

Supplementary Figure S8. Unrooted phylogenetic tree of the predicted *Acidihalobacter* Carbonic anhydrase (CA, *can*) amino acid sequences and their best hits from the NCBI non-redundant database. *Acidihalobacter* CA proteins are colored red, Chromatiales proteins are colored green and other acidophiles proteins are colored blue. The time scale bar represents 0.1 amino acid substitution per site.

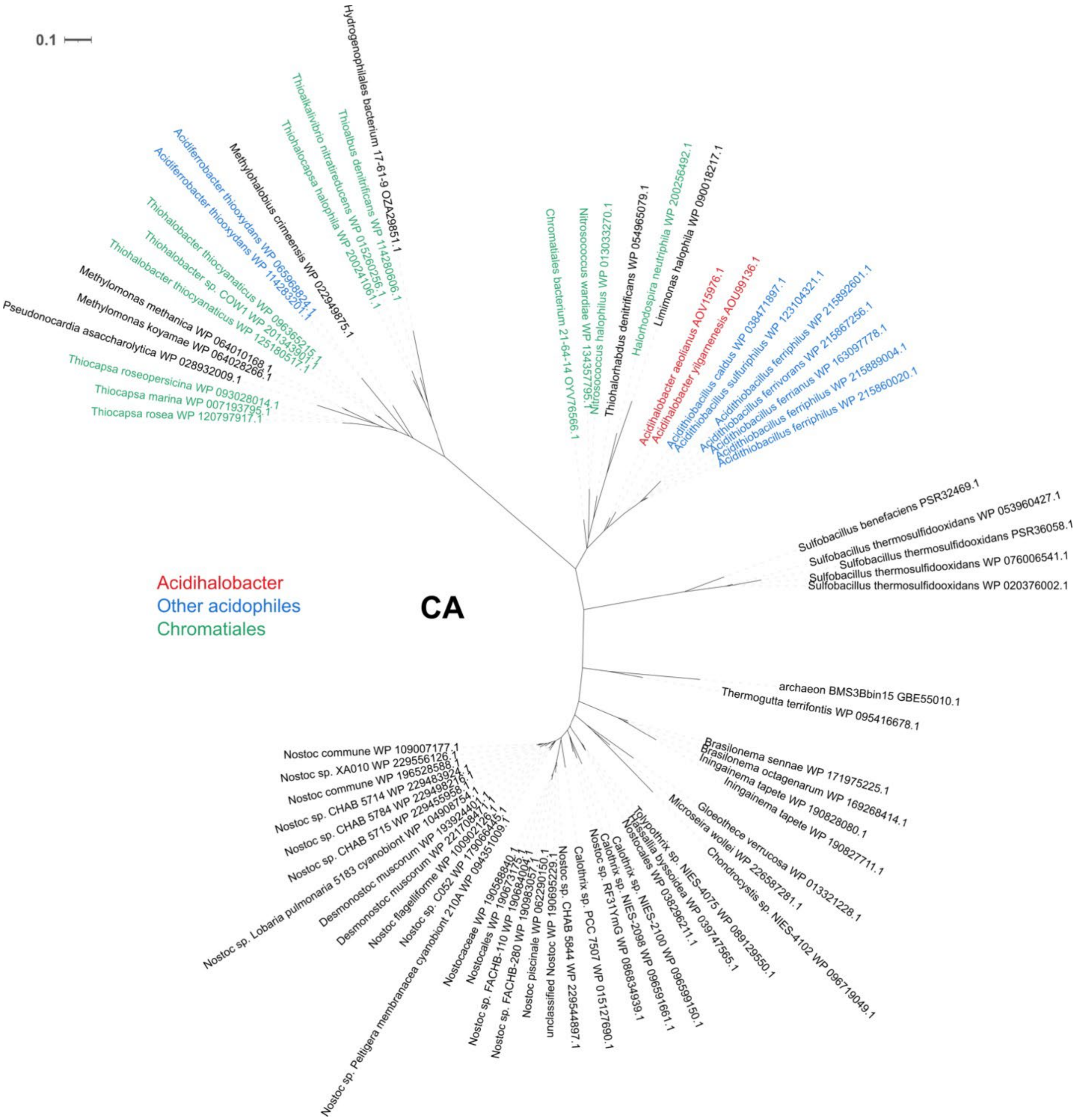

Supplementary Figure S9. Unrooted phylogenetic tree of the predicted *Acidihalobacter* ClcA amino acid sequences and their best hits from the NCBI non-redundant database. *Acidihalobacter* ClcA proteins are colored red, Chromatiales proteins are colored green and other acidophiles proteins are colored blue. The time scale bar represents 0.1 amino acid substitution per site.

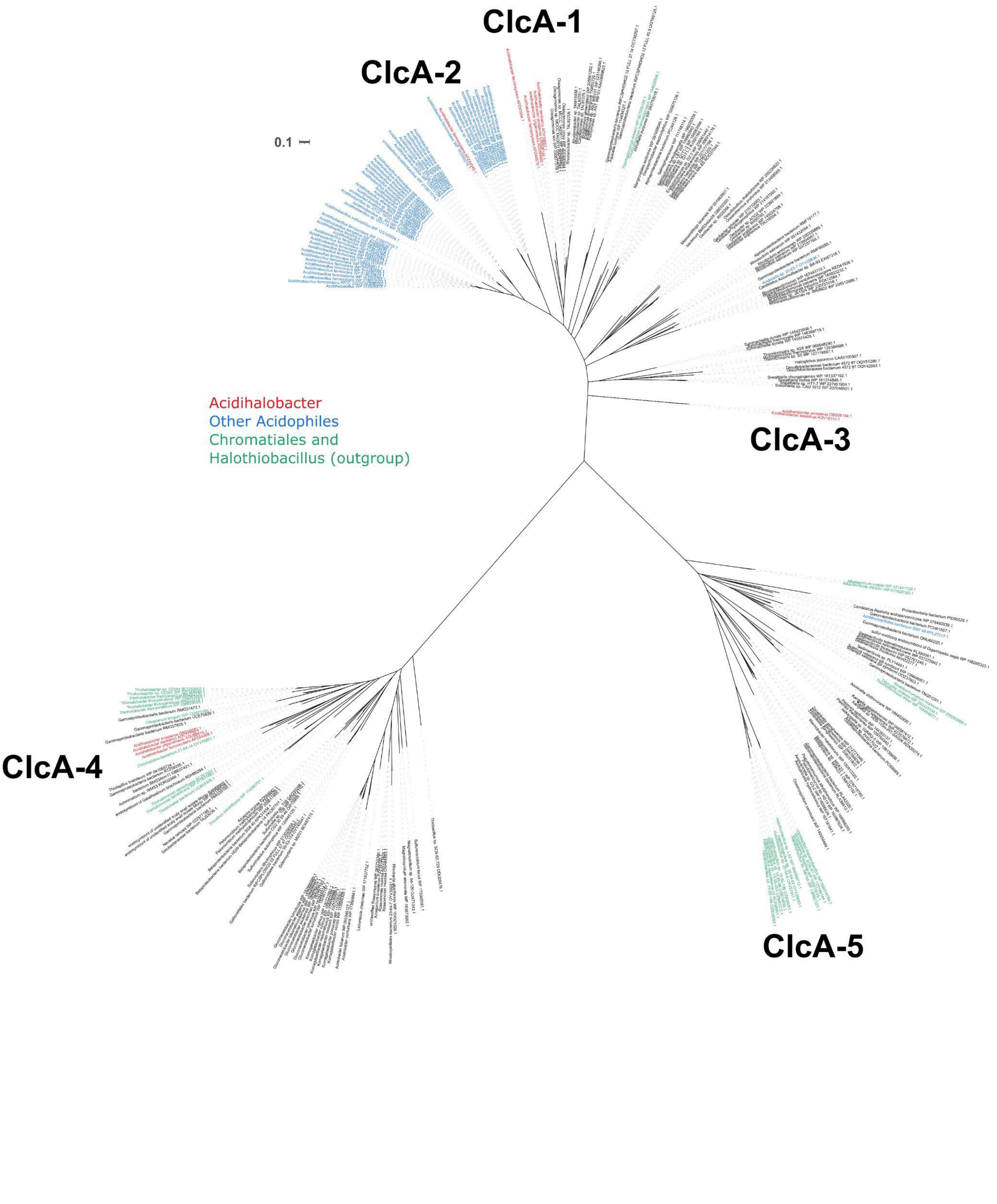

Supplementary Figure S10. Genomic context of *clc*A-4 genes in the *Acidihalobacter* genomes and *H. neapolitanus*. Grey background shows synteny between genomes; Red= involved in acid and osmotic tolerance, blue= other genes.

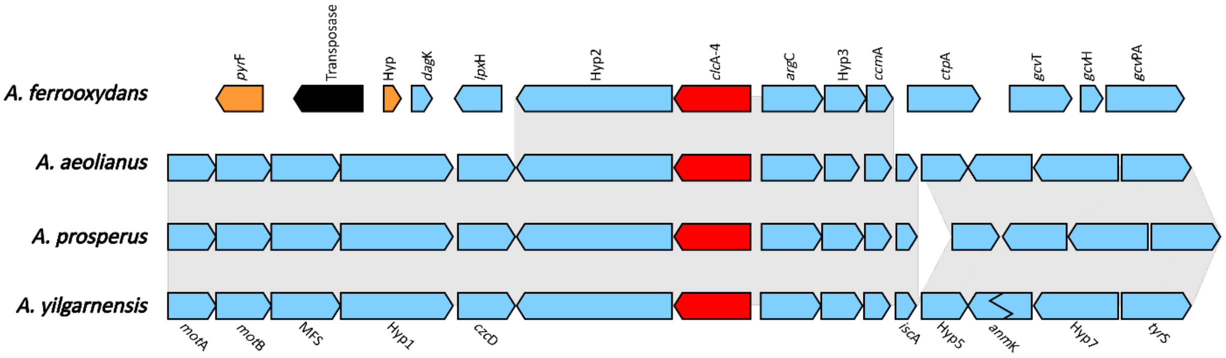

Supplement: Supplementary file 1 [file Data_Sheet_1.PDF]
